# Scale matters: risk perception, return expectations, and investment propensity under different scalings

**DOI:** 10.1007/s10683-018-09598-4

**Published:** 2018-12-01

**Authors:** Christoph Huber, Jürgen Huber

**Affiliations:** Department of Banking and Finance, University of Innsbruck, Universitätsstrasse 15, 6020 Innsbruck, Austria

**Keywords:** Behavioral finance, Judgment, Risk perception, Scaling, Presentation format, D14, D18, G11, G41

## Abstract

With a novel experimental design we investigate whether risk perception, return expectations, and investment propensity are influenced by the scale of the vertical axis in charts. We explore this for two presentation formats, namely return charts and price charts, where we depict low- and high-volatility assets with distinct trends. We find that varying the scale strongly affects people’s risk perception, as a narrower scale of the vertical axis leads to significantly higher perceived riskiness of an asset even if the underlying volatility is the same. Furthermore, past returns predict future return expectations almost perfectly. In our setting perceived profitability was considered more important than perceived riskiness when making investment choices. Overall we show that adapting the scale of a chart makes it easier to recognize yearly return variations *within* a single security, but at the same time makes it harder to identify differences *between* dissimilar securities. This is something regulators should be aware of and take into account in the rules they set.

**Electronic supplementary material:**

The online version of this article (10.1007/s10683-018-09598-4) contains supplementary material, which is available to authorized users.

## Introduction

When Gulliver traveled to Lilliput he was a giant. On his next journey to Brobdingnag he was a dwarf. While he had not changed, the scale of everything around him had. It seems that the scale we see something in plays a major role in how we perceive it. In financial practice, the scaling of price and return charts, e.g. in documents given to customers, is an important issue—recognized by practitioners, but mostly ignored by regulators and research so far. We mention regulators as, for example, the European Union sets rules for the presentation of a security’s past performance in a Key Investor Information Document (KIID; see Commission Regulation (EU) No 583/2010). According to that regulation, returns have to be shown in the form of bar graphs with a linear vertical axis. Additionally, the scale has to be adapted appropriately and *shall not compress the bars so as to make fluctuations in returns harder to distinguish* (p. 15). While the European Commission sees the potential problems of highly compressed bars, it remains unclear what consequences arise regarding the risk and profit expectations to-be-identified by investors, and hence, regarding investment decisions. Maximizing the return bars on the available space makes yearly fluctuations more distinguishable, but also brings the danger of misinterpretation of the returns as highly volatile and therefore highly risky, even when they are not. Compressing the bars, however, could lead to risk being perceived as too low, possibly exposing consumers to unexpectedly high losses. The fast emergence of robo-advisers, online brokers, and new products like e.g. cryptocurrencies add to the importance of gaining a better understanding of how people’s risk perception and investment propensity are influenced by different graphical representations of past returns.

As individuals focus on graphical and salient pieces of information in their information processing strategies (Jarvenpaa 1989), there is a wide range of literature on graphical representations of financial time series. One strand of research tackles the question of which presentation formats (e.g. returns, prices, or distributions) increase potential investors’ forecasting abilities and accuracy. Return charts are associated with lower expected returns (Glaser et al. 2018) but also with higher perceived uncertainty (Diacon and Hasseldine 2007), compared to price charts. Stössel and Meier (2015) also discuss framing effects of different presentation formats on risk perception, but restrict themselves to different forms of graphical representations in the narrow domain of the KIID.^1^ While Weber et al. (2005) find no significant improvement in perceived risk with continuous density distributions, Kaufmann et al. (2013) and Ehm et al. (2014) develop possibilities to better calibrate people’s risk perception by experience sampling from return distributions. However, these efforts require and imply a known stochastic process underlying the financial instrument to be assessed. In real-world applications, however, we have to rely on historical data, which may or may not give a good estimate for future returns and volatilities.

A number of studies has investigated graphical distortions in information processing, most notably regarding corporate reports (see e.g. Beattie and Jones 1992). They show that a disproportionate representation of the underlying data can be misleading (Tufte 1983) and is often purposely used to create a more favorable view.^2^ However, only few studies have investigated potential effects of varying a graph’s vertical axis scale without violating proportionality principles:^3^ Cleveland et al. (1988) examine the ‘shape parameter’ of graphs—that is, the ratio of the horizontal and vertical distances spanned by the data, while holding the scale’s range constant. Lawrence and O’Connor (1992, 1993) examine scale effects with regard to people’s forecasting ability in financial time series. They find that large scales or high variability in the presented time series leads to overly narrow confidence intervals. To our knowledge, however, the vertical axis scale’s relevance towards risk communication and investment decisions has not yet been investigated.

Our aim with this paper is to fill this gap by providing a systematic and rich analysis of the scale effect in graphical representations of financial time series. The research question we address is whether the presentation scale—narrow or wide—affects people’s risk perception, return expectations, and propensity to invest. We define a chart as having a *narrow* scale when the time series depicted extends close to the upper or lower borders of the chart, while a *wide* scale leaves ample space above and below.

To explore our research question we conduct a laboratory experiment with a 2 × 2 design where we vary the presentation scale (narrow or wide) and the presentation format: assets are presented either as return bar charts or as price line charts. In a within-subjects design we ask participants to assess the riskiness, expected return, and attractiveness as investment of the assets. In a second task subjects make pairwise comparisons between these assets along the same three dimensions.

We find that varying the scale strongly affects people’s risk perception, namely, that a narrower scale of the vertical axis leads to significantly higher perceived riskiness of an asset across price and return charts, even if the underlying volatility is the same. We demonstrate that adapting the scale to the span of the bars is reasonable with regard to recognizing yearly return variations *within* a single security, but at the same time makes it harder to identify differences *between* dissimilar securities. This result is robust for different historical return trends. We further find that past returns predict future return expectations almost perfectly irrespective of the scale. Risk perception is highly correlated with losses which in turn drive investment behavior. Concerning investment choices, subjects tend to invest in the asset they regard as more profitable, even if they think it bears higher risk.

This study extends the existing literature in several important ways: We analyze previously unexplored scale effects in a systematic and clean experimental setup; we embed these issues directly into the context of information presentation in financial markets; and we explore different aspects of financial decision-making relating to the scale, presentation format, and underlying asset fundamentals in individual assessments as well as in pairwise comparisons.

We think our findings are also informative for regulators: As we show, adapting the scale of a chart makes it easier to recognize yearly return variations *within* a single security, but at the same time makes it harder to identify differences *between* dissimilar securities. Regulators should be aware of—and attentive to—the potentially distorting effects of different axis scales in performance charts. While return bar charts are appropriate, allowing issuers to adapt the axis scale arbitrarily leaves room for deliberate action aimed at distorting investors’ perceptions about risk. Keeping the presentation scale constant across different securities enables better identification of risk and therefore easier comparisons.

## The experiment

### Returns and prices

To systematically vary expected return, time trend, and volatility of percentage return time series we create eight distinct return paths consisting of ten (hypothetical) annual returns each. Each return path consists of a deterministic trend (positive stable, negative stable, increasing, or decreasing) and a normally distributed noise term $\varepsilon _t \sim N (\mu , \sigma ^2)$ with $\mu = 0.0\%$, $\sigma ^2 = 1.4\%$ and $t=1,...10$. Low-volatility assets consist of a linear return path plus the noise term for each year *t*. High-volatility assets have the same linear return paths but with the noise term multiplied by 6 before it is added.

**Fig. 1:**
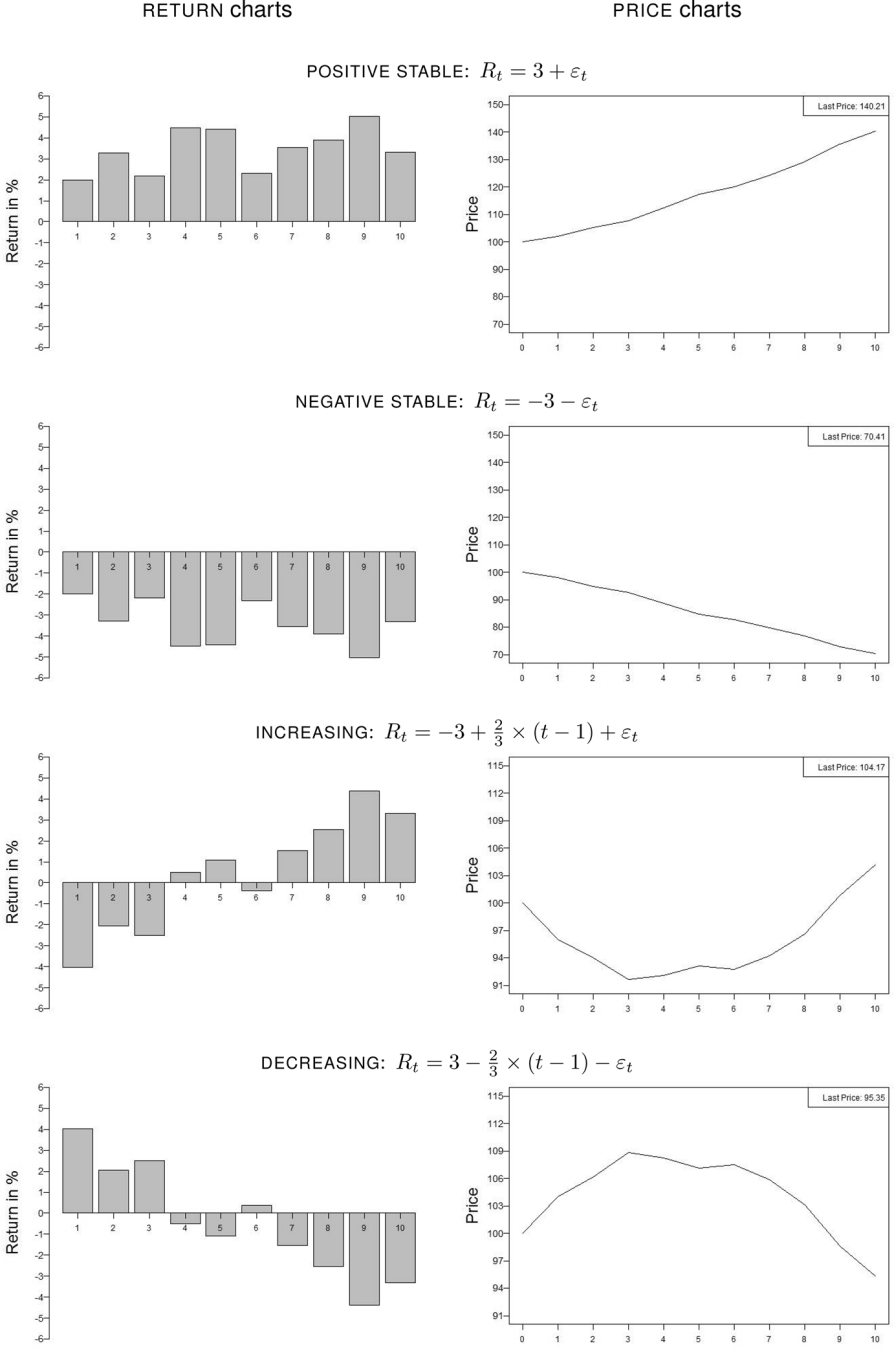
Return and price paths. This figure shows the four distinct return trends with the low volatility level as a function of time, depicted as return bar charts (left) and price line charts (right). For high-volatility assets, the added error term $\varepsilon _t$ is multiplied by six

Figure 1 shows each distinct return trend as a function of time, depicted as ${\textsc {return}}$ charts (left) and ${\textsc {price}}$ charts (right). Assets with a ${\textsc {positive stable}}$ trend are set up to yield positive returns fluctuating around a mean of 3% per year. The return path ${\textsc {increasing}}$ starts at − 3% in the first year and linearly increases to + 3% in the tenth year plus a noise term. Assets with trend negative stable contain the returns of the asset with trend positive stable multiplied by − 1. Analogously returns in trends decreasing are the returns of trend increasing multiplied by − 1. Price paths are generated by successively applying the corresponding returns to an initial price of 100.

### Experimental tasks

In the experiment subjects have to complete two main tasks, Task I and Task II. In both tasks participants were instructed to suppose that they want to invest 5000 euros. Subjects are then presented with charts of hypothetical assets and are asked to assess the respective riskiness and profitability of one asset at a time in Task I, and to compare two assets at a time along these dimensions in Task II. There are two variants for each task: either a return bar representation (${\textsc {return}}$) or a line chart depicting the price development (${\textsc {price}}$).

Task I consists of a 2 × 2 treatment design in which we vary the presentation format (${\textsc {return}}$ or ${\textsc {price}}$) and the presentation scale of the vertical axis (${\textsc {narrow}}$ or ${\textsc {wide}}$) to identify these variables’ effect on risk perception as well as on return expectations and investment propensity. Subjects sequentially see eight different paths (${\textsc {return}}$ or ${\textsc {price}}$) and have to assess the assets’ riskiness and estimate its returns over the following year and over the next five years.^4^ Whenever subjects are presented with return charts they are explicitly asked about future returns; when they see price charts they are asked to estimate future prices.^5^ Each participant was presented with eight out of 16 possible return (price) charts (eight different assets in two different presentation formats, ${\textsc {narrow}}$ and ${\textsc {wide}}$) in which each chart has the same probability of appearing. The order in which participants saw the assets was randomized and participants were not aware of being presented only with a selection of the possible assets.

In Task II subjects make pairwise comparisons between assets regarding their riskiness and expected return. In a 2 × 2 design the combinations of volatility and scale of the vertical axis are varied in four distinct conditions: same scale/same volatility, same scale/different volatility, different scale/same volatility, and different scale/different volatility. Except for Condition ${\textsc {same}}$ (same scale and same volatility), we name each condition after the variable in which the two charts of a pair *differ*. With this set-up we are able to generate a distinct number of 16 pairs for Condition ${\textsc {same}}$,^6^ four pairs for Condition volatility, and eight pairs each for conditions scale and both. Subjects are presented with a total of eight randomly chosen pairs—two for each condition. In this task subjects have to compare two assets at a time. They are asked to decide which of the two assets they perceive as riskier; which asset they think is more profitable; and which asset they would rather invest in. For each question there is also the possibility to choose the neutral option ‘the same for both’ (later also referred to as ‘indifferent’).

**Fig. 2:**
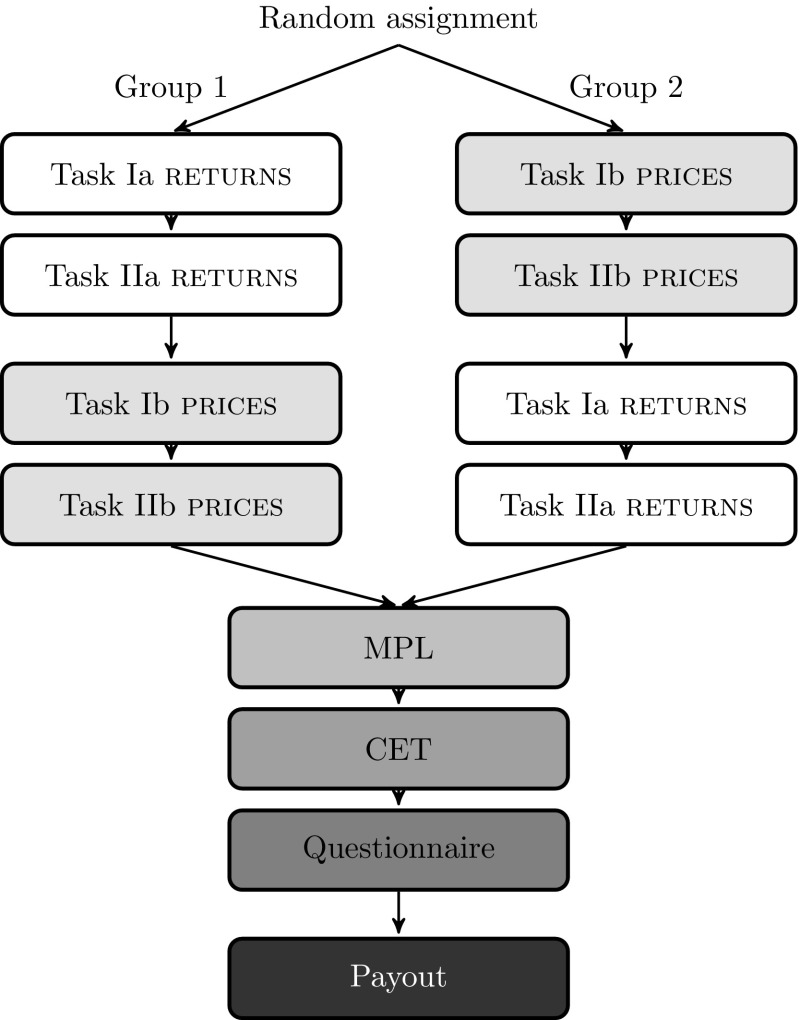
Graphical overview of the experimental procedure. Subjects are randomly assigned into two groups with Group 1 being presented with ${\textsc {return}}$ charts first (Tasks Ia and IIa) and ${\textsc {price}}$ charts second (Tasks Ib and IIb) and Group 2 vice versa. Both groups complete a multiple price list task (MPL) and a certainty equivalence task (CET) to elicit risk and loss aversion parameters, as well as a questionnaire after the experiment

In total, 32 charts have been considered: 4 trends × 2 volatilities (high/low) × 2 formats (return/price) × 2 scales (wide/narrow) = 32; 16 price charts and 16 return charts. For both tasks there are two variants: In Tasks Ia and IIa subjects are presented with return charts, in Tasks Ib and IIb they see price charts. In Task I, each subject considers eight random return charts (Ia) and eight random price charts (Ib), and in Task II each subject considers eight random return chart comparisons (IIa) and eight random price chart comparisons (IIb). Hence, each subject sees a potentially different selection of charts. The order is randomly determined and subjects are randomly assigned to one of two groups to eliminate any order effects (see Fig. 2 for the timeline and the two possible sequences).

**Fig. 3:**
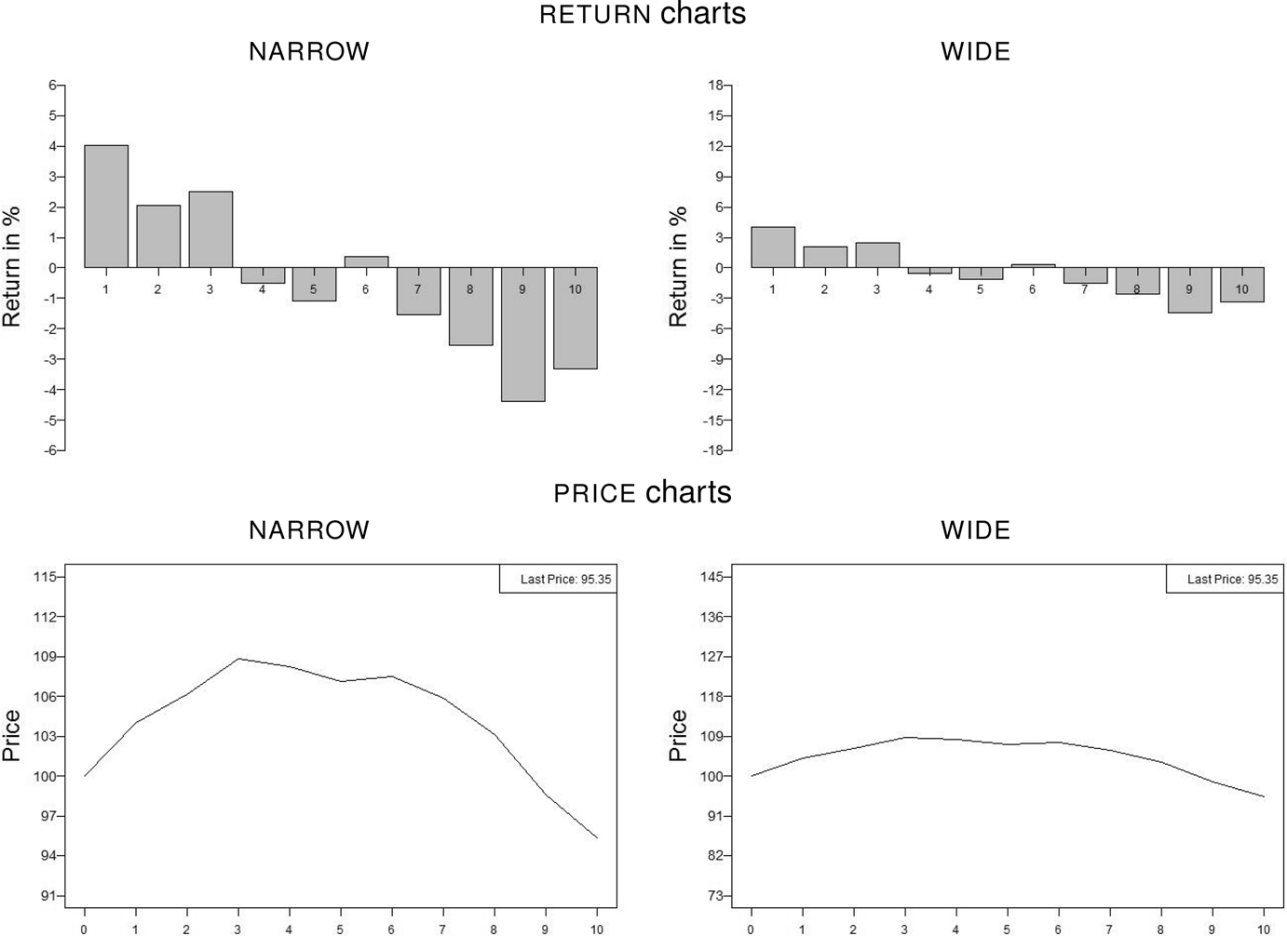
Exemplary representations of the ${\textsc {low}}$-volatility asset with trend ${\textsc {decreasing}}$ in a ${\textsc {return}}$ chart (top) and a ${\textsc {price}}$ chart (bottom) for presentation scales ${\textsc {narrow}}$ (left) and ${\textsc {wide}}$ (right). For return charts the value zero and for price charts the initial price of 100 as well as each tick are at the same positions for both scales. In return bar representations the tick size on a ${\textsc {wide}}$ scale is three times the one on a ${\textsc {narrow}}$ scale; tick sizes in price representations are adjusted accordingly

In both presentation formats we vary the scale of the vertical axis to create a ${\textsc {narrow}}$ and a ${\textsc {wide}}$ representation of each asset’s past performance. In return charts, the maximum value on the vertical axis and the tick size of ${\textsc {wide}}$-scaled representations are three times the corresponding values of representations with scale ${\textsc {narrow}}$. For price charts the scales are adapted analogously. Figure 3 shows an example of ${\textsc {return}}$ charts (top) and ${\textsc {price}}$ charts (bottom), each with presentation scales ${\textsc {narrow}}$ (left) and ${\textsc {wide}}$ (right). In each return (price) chart the value zero (100) as well as each tick (with precise values depending on asset and scale) is at exactly the same position in the graph to maintain consistency. Additionally, in order to reduce noise in estimating prices we provide the last price in the upper right corner of price charts (Glaser et al. 2018). In the instructions, we explicitly point out that the scale of the vertical axis might change over the course of the experiment. This note also appears when subjects review the on-screen instructions at any point in time during an experimental task. This prominently-placed reminder should ensure that our results are not driven by subjects’ inattention to the scale. To guarantee subjects’ understanding of the term ‘return’ we also include a definition stating that the return is defined as the percentage change of the price over one year. Table 1 summarizes the asset- and chart-specific variables and respective options: each chart is a distinct combination of volatility, trend, presentation format, and scale.

**Table 1:** Summary of variables in each performance chart

	Variable	Possible options
Asset specific	Volatility	${\textsc {low}}$ or ${\textsc {high}}$
	Trend	${\textsc {positive stable}}$,
		$${\textsc {negative stable}}$$,
		$${\textsc {increasing}}$$,
		or ${\textsc {decreasing}}$
Chart specific	Presentation format	${\textsc {return}}$ or ${\textsc {price}}$
	Scale (vertical axis)	${\textsc {wide}}$ or ${\textsc {narrow}}$

This table summarizes the relevant variables in specific to assets and charts: the volatility and trend of an asset, and the presentation format and scale of a chart

### Implementation of the experiment

We conducted nine experimental sessions with a total of 193 students of business administration or economics in May and June 2017 at the Innsbruck EconLab at the University of Innsbruck. The experiment was programmed and conducted using oTree by Chen et al. (2016). Subjects were recruited with hroot by Bock et al. (2014). 45% of subjects were female; the mean age was 23; and about 51% of subjects had completed an undergraduate course in financial management.

In total, each session lasted approximately 40 min. This included studying on-screen instructions for each part of the experiment as well as a multiple price list task measuring subjects’ risk attitudes (Holt and Laury 2002) and a certainty equivalence task to assess loss aversion (Gächter et al. 2010) using oTree applications by Holzmeister (2017). After the main experiment subjects completed a questionnaire assessing their risk attitudes and demographics. A graphical overview of the experimental procedure, as well as the experimental instructions, screenshots of the decision tasks, and exemplary charts for each condition are provided in Online Appendices C, D, and E.

Subjects are incentivized by being paid one randomly chosen return of the asset they chose to rather invest in in one randomly chosen pair they were presented with for both parts (prices and returns) of Task II.^7^ The chosen return times two is added to an initial amount of 5 euros for each task. For example, if the chosen asset of the randomly drawn pair pays 10% in the randomly drawn year, the participant receives 5 euros $\times \, (1 + 2 \times 0.10) = 6$ euros for this task. Total payouts varied between 6.30 euros and 16.30 euros with a mean of 11.65 euros; these include payouts from the risk and loss aversion tasks.

## Results from Task I: individual assessments

We organize the presentation of results as follows: first we analyze subjects’ individual assessments (Task I), starting with perceived risk, followed by expected returns and investment propensity. Subsequently, the same structure is repeated for the analysis of pairwise comparisons (Task II).

### Risk perception in individual assessments

We start our discussion by examining the influence of the scaling of the vertical axis on risk perception. We present analyses along the following dimensions: for both presentation formats (${\textsc {return}}$ and ${\textsc {price}}$ charts) we compare the influence of scaling of the vertical axis (${\textsc {wide}}$ vs. ${\textsc {narrow}}$). To get a comprehensive picture we do this separately for the four different return trends (${\textsc {positive stable}}$, ${\textsc {negative stable}}$, ${\textsc {increasing}}$, and ${\textsc {decreasing}}$), where we have each return trend once with a low and once with high level of return volatility (${\textsc {low}}$ or ${\textsc {high}}$).

**Fig. 4:**
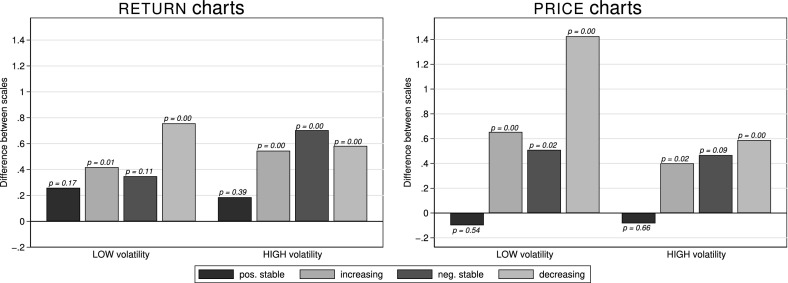
Differences in average perceived risk (in NARROW minus WIDE) by trend and scale presented as ${\textsc {return}}$ charts (left) and ${\textsc {price}}$ charts (right). This figure depicts differences in average perceived risk (on a scale from 1 = “not risky at all” to 7 = “very risky”) for ${\textsc {return}}$ chart and ${\textsc {price}}$ chart representations of ${\textsc {low}}$ (left bars in each panel) and ${\textsc {high}}$ (right bars in each panel) volatility assets. *p*-values above the bars are from Fisher-Pitman permutation tests on the subject-demeaned data. Each of the sixteen bars summarizes between 179 and 206 observations

Figure 4 shows the differences in average perceived risk (elicited on a scale from 1 to 7) for each asset from ${\textsc {return}}$ charts (left panel) and ${\textsc {price}}$ charts (right panel). The differences are from the same asset being displayed once with a ${\textsc {wide}}$ and once with a ${\textsc {narrow}}$ scale.^8^ The four bars in each group of bars represent the four different trends; ${\textsc {low}}$ volatility is shown in the left group of each panel while ${\textsc {high}}$ volatility is shown in the right group of each panel.

*Result 1* In individual assessments assets are perceived as riskier when presented on a ${\textsc {narrow}}$ scale than when presented on a ${\textsc {wide}}$ scale.

*Support* For all assets, except those with a ${\textsc {positive stable}}$ trend, a ${\textsc {narrow}}$ scale leads to higher perceived risk compared to a ${\textsc {wide}}$ scale. This holds for both presentation formats and both volatility levels. To test the statistical significance of the differences between charts with ${\textsc {narrow}}$ and ${\textsc {wide}}$ axis scales, we run Fisher-Pitman permutation tests on the subject-demeaned data.^9^ 10 out of 12 tests for assets other than with a ${\textsc {positive stable}}$ trend deliver *p*-values of 0.02 or smaller (the remaining two having *p*-values of 0.09 and 0.11, respectively), corroborating that assets are perceived riskier when presented on a ${\textsc {narrow}}$ scale—we conjecture that this is the case because fluctuations are easier visible on a ${\textsc {narrow}}$ scale. As the ${\textsc {positive stable}}$ trends show the lowest overall risk perceptions we conjecture that the non-difference in their perceived riskiness results from the fact that these always-positive returns (monotonically increasing prices, respectively) are never perceived as risky, no matter how they are displayed or whether they fluctuate more.

As we prominently make subjects aware of varying axis scales in the instructions, and as we find no relationship between the differences in risk perception and the time it took subjects to complete all related tasks,^10^ we attribute the reported differences in perceived risk to the differences in axis scales, as the differences seem not to stem from subjects being unaware of the varying axes or inattentively clicking through the tasks.

Regarding different return paths we observe that the ${\textsc {positive stable}}$ trend is seen as least risky with average assessments between 2.08 and 3.51 on a 7-point scale, whereas ${\textsc {negative stable}}$ and ${\textsc {decreasing}}$ trends are viewed as carrying the highest risk (average riskiness assessments between 4.52 and 5.94) with trend ${\textsc {increasing}}$ being in-between across all presentation formats.^11^ This implies that an asset’s volatility (standard deviation of returns) does not necessarily determine people’s perceptions about its risk: e.g., for trend ${\textsc {negative stable}}$ we find no difference in perceived risk between the ${\textsc {low}}$- and ${\textsc {high}}$-volatility assets in return and price charts ($p=0.17$ and $p=0.40$)—even though their volatility differs by a factor of six. Furthermore, the standard deviation is the same for ${\textsc {negative stable}}$ and ${\textsc {positive stable}}$, but they are at opposite ends regarding perceived riskiness. This shows that subjects perceive losses (negative returns) as *risk*, while profits are perceived as not risky, even if they vary as much as losses do.^12^

Additionally, one remarkable side result with potentially important implications for practitioners and regulators is that people perceive risk as significantly higher when presented with ${\textsc {return}}$ charts as compared to ${\textsc {price}}$ charts (five out of eight *p*-values are significant at $p<0.01$; all differences have the same sign; details are provided in Online Appendix B).

### Expected returns in individual assessments

**Fig. 5:**
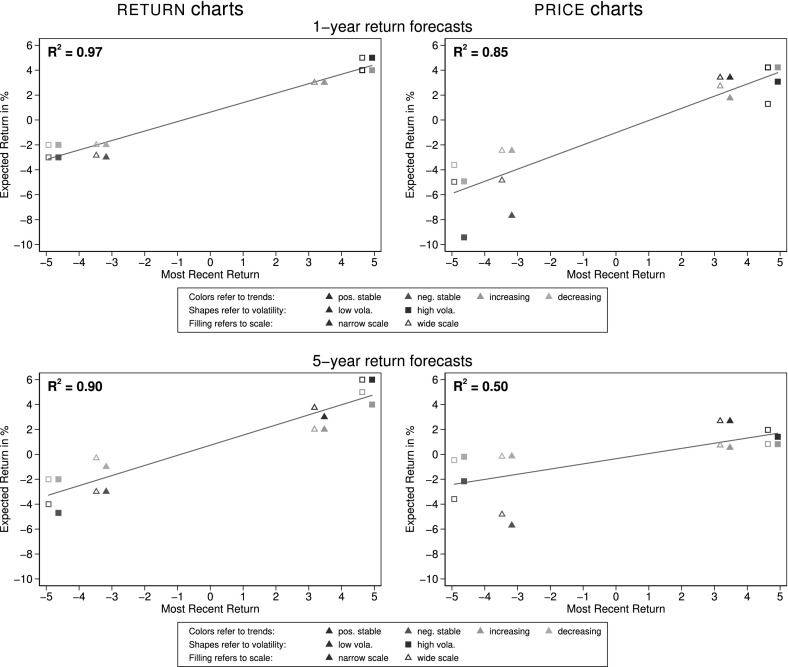
Median one-year and five-year return forecasts. This figure shows the median one-year (upper panel) and five-year (lower panel) return forecasts as a function of the most recent return, i.e. the return in Year 10. For better visibility, i.e. to avoid overlapping medians, we add 0.15% to the most recent return of scaling ${\textsc {narrow}}$ and deduct 0.15% for those of scaling ${\textsc {wide}}$ on the horizontal axis. Each point represents the median of between 83 and 109 observations

Besides eliciting subjects’ perceptions about risk we also asked participants to enter point estimates of future returns (when ${\textsc {return}}$ charts were shown) or future prices (when ${\textsc {price}}$ charts were shown) for a shorter (one year) and a longer (five year) horizon.^13^ We discuss short-term forecasts first. The upper panels of Fig. 5 depict the median one-year-ahead return expectations for each asset (vertical axis) in relation to the last return (horizontal axis) in both presentation formats for scales ${\textsc {wide}}$ and ${\textsc {narrow}}$.

*Result 2* Expected returns are driven by the latest return. We find no systematic influence of the scale on return expectations in individual assessments.

*Support* Short-term return forecasts are not the same across assets, but strongly depend on the last return. Subjects thus seem to behave as short-term trend-followers. With an $R^2$ of 0.97 and a slope of 0.76 the past return almost perfectly explains return predictions when returns are shown (upper-left panel of Fig. 5). When prices are shown (right panel) there is more dispersion, especially when the last return is negative. Still, with a slope of 0.98 and a $R^2$ of 0.85 the last return is a very good predictor of expected returns. This is consistent with Grosshans and Zeisberger (2018), who analyze forecasts for price paths similar to the ${\textsc {increasing}}$ and ${\textsc {decreasing}}$ trends in the present study, as they also report strong beliefs in short-term trend continuations. In both presentation formats we do not find a systematic influence of the scale (${\textsc {narrow}}$ or ${\textsc {wide}}$).

We also asked subjects for their five-year return prediction (return per year); respectively price prediction (price in five years). For returns we find a very similar and consistent pattern to the one-year-predictions where the last return is again a very good predictor with a slope of 0.82 and an $R^2$ of 0.90 (see lower panels of Fig. 5). Returns calculated from ${\textsc {price}}$ predictions also show a strong positive relation between last return and expected return. However, with a slope of 0.42 and an $R^2$ of 0.50 the relation is markedly flatter and weaker than for the one-year price data or the ${\textsc {return}}$ data. In contrast to Glaser et al. (2007) we find that even for five-year-ahead forecasts, on average participants do not expect trend reversals to the extent of a change in signs—we find that the slope calculated from prices is only half as steep as for the one-year forecasts.

### Investment preferences in individual assessments

We elicit subjects’ propensities to invest (on a scale from 1 to 7) for each of the displayed return and price charts. We find these to be negatively related to perceived riskiness,^14^ i.e. assets with a ${\textsc {positive stable}}$ trend are the ones subjects would most like to invest in, while those with ${\textsc {negative stable}}$ trends are least preferred. What we are interested in, however, is, whether there are differences in investment preferences between scales (${\textsc {narrow}}$ vs. ${\textsc {wide}}$), i.e. whether the differences in risk perception we report in Section 3.1 translate into differences in investment propensities.

*Result 3* Investment propensity is driven by an asset’s historical return and volatility as well as by subjective risk perception and expected returns. Varying the scale, however, has almost no influence on investment propensity.

**Fig. 6:**
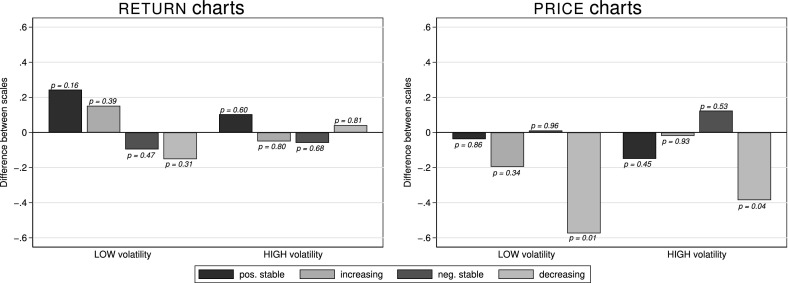
Differences in average investment propensity (in NARROW minus WIDE) by trend and scale presented as RETURN charts (left) and PRICE charts (right). This figure depicts differences in average investment propensity (on a scale from 1 = “very unlikely to invest” to 7 = “very likely to invest”) for ${\textsc {return}}$ chart and ${\textsc {price}}$ chart representations of ${\textsc {low}}$ (left bars in each panel) and ${\textsc {high}}$ (right bars in each panel) volatility assets. *p*-values above the bars are from Fisher-Pitman permutation tests on the subject-demeaned data. Each of the sixteen bars summarizes between 179 and 206 observations

*Support* Fig. 6 summarizes subjects’ answers by displaying the differences in average investment propensity (value in ${\textsc {narrow}}$ minus value in ${\textsc {wide}}$) by trend and scale presented as ${\textsc {return}}$ charts (left) and ${\textsc {price}}$ charts (right). We only find a significant difference for ${\textsc {decreasing}}$ trends in ${\textsc {price}}$ charts, i.e. there is a higher likelihood to invest when these are displayed with a ${\textsc {wide}}$ scaling, while the other 14 tests do not yield significant differences. Hence, we do not find large, systematic differences between scales regarding subjects’ investment propensity.^15^

To explain investment behavior more comprehensively, we estimate least squares regressions similar to Nosić and Weber (2010, also see Sarin and Weber 1993; Jia et al. 1999). The estimates suggest that besides an asset’s historical return and volatility, lower perceived risk of an asset (given a specific presentation format) and especially higher long-term expected returns increase the likelihood of investing, confirming the intuition that investment propensity substantially relies on people’s subjective assessments.^16^

## Results from Task II: pairwise comparisons

In Task II subjects are asked to compare two assets displayed on the screen at the same time. We ask for perceived riskiness (“*Which of the two assets do you consider to be more risky?*”), perceived profitability (“*Which of the two assets do you consider to be more profitable?*”), and investment propensity (“*In which of the two assets would you rather invest?*”). In four different conditions the two displayed assets vary by neither scale nor volatility (Condition ${\textsc {same}}$), only in the scale of the vertical axis (${\textsc {scale}}$), only in volatility (${\textsc {volatility}}$), or by both (${\textsc {both}}$), respectively, but the two assets shown always share the same presentation format and trend. Subjects compare assets eight times with ${\textsc {return}}$ charts and eight times with ${\textsc {price}}$ charts. For an investment decision this pairwise comparison could be a more natural setting than Task I, as people often consider more than one investment possibility before deciding to invest in one particular financial instrument.

**Fig. 7:**
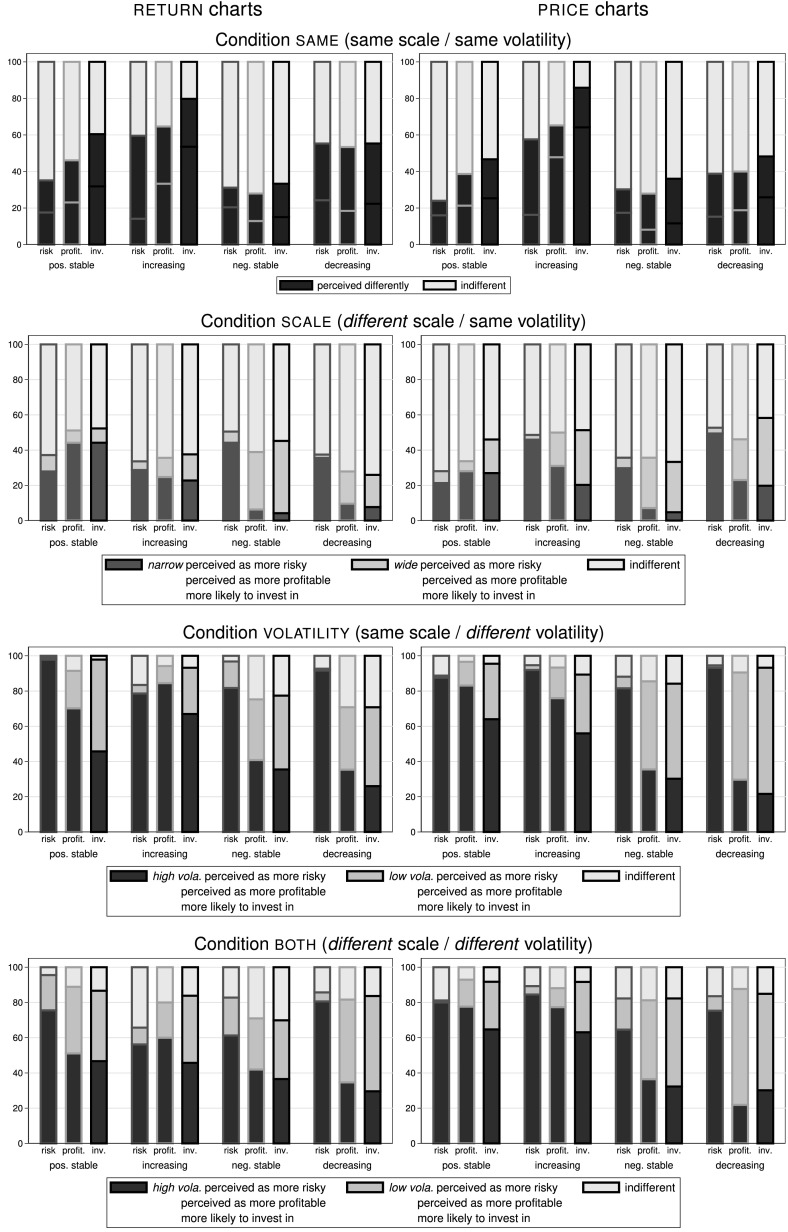
Perceived riskiness, perceived profitability, and investment propensity in Task II. This set of panels shows the percentage of decisions in which subjects perceive the riskiness (first bar in each set labelled ‘risk’) and the profitability (second bar; ’profit.’) the same or differently, and in which subjects are more likely to invest in (third bar; ’inv.’), between different scalings and volatilities. The left panels show data for ${\textsc {return}}$ charts, while the right panels show the respective data for ${\textsc {price}}$ charts. From top to bottom we show the four different conditions, where the condition name corresponds to the variable in which the two assets of a pair *differ*: ${\textsc {same}}$ (same scale / same volatility), ${\textsc {scale}}$ (different scale / same volatility), ${\textsc {volatility}}$ (same scale / different volatility), and ${\textsc {both}}$ (different scale / different volatility). In each panel data for the four distinct price trends are shown separately. Each of the eight panels summarizes between 314 and 386 observations for each variable

Figure 7 summarizes the results of Task II for ${\textsc {return}}$ charts (left panels) and ${\textsc {price}}$ charts (right panels). Each panel shows from left to right the four distinct trends (${\textsc {positive stable}}$, ${\textsc {increasing}}$, ${\textsc {negative stable}}$, and ${\textsc {decreasing}}$) and from top to bottom the four conditions ${\textsc {same}}$, ${\textsc {scale}}$, ${\textsc {volatility}}$, and ${\textsc {both}}$. The first bar of each group of bars within a panel always corresponds to perceived riskiness (‘risk’), the second bar to perceived profitability (‘profit.’), and the third bar to investment propensity (‘inv.’). Each bar shows the percentage of decisions in which subjects perceive the assets as the same (light grey) or differently (black in the top row of panels; dark and light red in the second row for ${\textsc {narrow}}$ vs. ${\textsc {wide}}$ scaling, and dark vs. light blue in the bottom two rows for the high- vs. low-volatility assets).

### Risk perception in pairwise comparisons

*Result 4* Different scaling can distort people’s perception about an asset’s risk in pairwise comparisons of assets with the same volatility, as assets shown on a ${\textsc {narrow}}$ scale are perceived as more risky.

*Support* From the first row of panels in Fig. 7 we see that even when the two displayed assets have the same volatility and there is no difference in the scale, a surprisingly high share of between 24% and 60% of subjects perceive risk differently between the two assets. The share who perceives the risk differently is significantly higher for ${\textsc {increasing}}$ and ${\textsc {decreasing}}$ trends than for ${\textsc {positive stable}}$ and ${\textsc {negative stable}}$ trends ($p<0.01$).^17^ This holds for ${\textsc {return}}$ as well as ${\textsc {price}}$ charts. Probably subjects saw the similarity between the two charts/price paths shown, but thought there must be some difference to find and hence considered the two paths as differently risky. Data supporting this line of argumentation is the time subjects needed until they reached a decision: even though the scales are all equal (and thus easiest to compare) in Condition ${\textsc {same}}$, subjects took significantly longer here (25.02 sec. on average) than in any other condition (between 21.74 and 23.35 sec.)

The second line of panels of Fig. 7 shows the distribution of choices for Condition ${\textsc {scale}}$, i.e. when the asset volatility is the same but the scale is different (${\textsc {narrow}}$ on one side, ${\textsc {wide}}$ on the other). One could argue that this is the ‘trap’ case where two identical assets are shown with different scaling to see whether subjects can be misled and thus perceive the asset shown with the narrower scale as more risky. This is indeed the case in 21% to 49% of all cases, while the asset with the wider scale is perceived as riskier in only 2% to 9% of all cases (in the remaining cases subjects are indifferent—most likely correctly seeing that only the scaling is different between both sides). Results are similar for ${\textsc {return}}$ and ${\textsc {price}}$ charts.

In ‘vertically’ comparing subjects’ risk assessments between conditions ${\textsc {same}}$ and ${\textsc {scale}}$, where we vary the scale while keeping volatility constant, we find no significant difference in the proportion of ‘correct’ choices (in the sense of being indifferent between two almost identical or identical assets, i.e. with the same volatility): the average rates are 58% and 60%, respectively.

*Result 5* Different scaling can distort people’s perception about an asset’s risk in pairwise comparisons of assets with different volatilities. Depicted with the same scale, subjects regard the more volatile asset as riskier; with differing scales, a considerable fraction erroneously perceives the less volatile asset as riskier or views them as equally risky.

*Support* In the third row of panels of Fig. 7 we present the case where the volatility of the two assets varies by a factor of six while the scale is the same (Condition ${\textsc {volatility}}$). Here, both assets are displayed on exactly the same axes and it should thus be comparatively easy to identify the more volatile asset and, if volatility is perceived as ’risk’, also to identify this asset as the riskier one. We find that with a ${\textsc {positive stable}}$ trend in ${\textsc {return}}$ charts, almost 100% of subjects do exactly that. We also report very high shares of 90% and above identifying the more volatile asset as the riskier one for ${\textsc {decreasing}}$ trends, both in ${\textsc {return}}$ and ${\textsc {price}}$ charts, and for ${\textsc {positive stable}}$ and ${\textsc {increasing}}$ trends in ${\textsc {price}}$ charts. For ${\textsc {increasing}}$ trends in ${\textsc {return}}$ charts we find 17% of subjects to be indifferent between the two assets—most likely as both trends start negative and then mostly increase, which is perceived as equally good, irrespective of volatility. An interesting case are the ${\textsc {negative stable}}$ trends, especially for ${\textsc {return}}$ (but also, to a lesser degree, for ${\textsc {price}}$) charts: here around 20% do not see the less volatile asset as the less risky one. For ${\textsc {return}}$ charts, 15% even consider the more volatile asset as less risky. We conjecture that this is the case as all returns in the low-volatility case are clearly negative—hence, an investor always loses with this asset. With high volatility, the dispersion of returns is much wider and thus the chance of earning a positive return is also higher. The corresponding asset is therefore perceived as less risky in about every sixth decision.

Finally, the fourth row of panels in Fig. 7 displays the choices in Condition ${\textsc {both}}$, where the shown assets differ in their volatility and additionally in their scaling. Note that in this condition, one side depicts a low-volatility asset and the other a high-volatility asset, hence different scales lead to both assets being displayed either on a ${\textsc {wide}}$ or on a ${\textsc {narrow}}$ scale with the respective bars having comparable magnitudes. Comparing the results in this condition to the ones in the third row of panels we see that the choices are now more dispersed. While the high-volatility asset is still perceived as the more risky one in the majority of cases (between 56% and 85% of cases), ‘indifferent’ (up to 34%) and a preference for the low-volatility asset (up to 22% of cases) are chosen markedly more frequently than when the scaling is the same. In this cognitively demanding condition, results between and within ${\textsc {return}}$ and ${\textsc {price}}$ charts vary more than in other conditions. In particular, with trend ${\textsc {positive stable}}$ almost 20% see the low-volatility asset as the riskier one in return charts but choose ‘indifferent’ in price charts. We conjecture that in both cases subjects are misled by the different scalings. For trend ${\textsc {increasing}}$, however, in a remarkably high share of 34% of decisions subjects are indifferent between the high- and low-volatility assets with ${\textsc {return}}$ charts (for ${\textsc {price}}$ charts the respective number is only 11%). We argue that for these subjects the main decision criterion is the clear upward trend in returns, while the details of the vertical axis scale and the exact values play a smaller role.

Assets with trend ${\textsc {negative stable}}$ (third group of bars) are again a different story: a significantly higher share of subjects picks the less volatile asset as the riskier one than in any other trend bar ${\textsc {positive stable}}$ with ${\textsc {return}}$ charts (all other $p<0.05$). This hints at losses being a driving force behind risk perception as all returns are negative in the low-volatility asset but not in the high-volatility one. Hence, in the ${\textsc {negative stable}}$ trend, having more volatile returns increases the chance that an investor could end up with a positive return.

Assuming that participants should always regard the high-volatility asset as more risky (which may not hold e.g. in trend ${\textsc {negative stable}}$), we can distinguish between ‘correct’ and ‘incorrect’ choices. Along this line we compare the proportion of the two between conditions ${\textsc {volatility}}$ and ${\textsc {both}}$—where the scale is either the same for both assets or adapted to the respective asset’s volatility. On average, we find that the percentage of ‘incorrect’ choices, in which either the two assets are regarded as being equally risky or the low-volatility asset is seen as riskier, is significantly lower in Condition ${\textsc {volatility}}$ (13%) than in Condition ${\textsc {both}}$ (29%, $p < 0.01$).^18^ Hence, adapting the scale to the returns’ magnitude leads to significantly more mistakes when assessing the riskiness of two assets with differing levels of volatility.

### Perceived profitability in pairwise comparisons

We now turn to the analysis of perceived profitability in pairwise comparisons. The respective proportions for this variable are depicted in the second bar of each group of bars in Fig. 7 (‘profit.’).

*Result 6* Scaling can distort people’s perception about an asset’s profitability in pairwise comparisons of assets with the same volatility. For trends ${\textsc {positive stable}}$ and ${\textsc {increasing}}$, assets shown on a ${\textsc {narrow}}$ scale are regarded as more profitable; for trends ${\textsc {negative stable}}$ and ${\textsc {decreasing}}$, the opposite holds.

*Support* In the top row of panels in Fig. 7, showing results for Condition ${\textsc {same}}$, between 28% and 65% of subjects state a difference in perceived profitability, even though the two assets are essentially identical. The patterns observed, both with ${\textsc {return}}$ charts (left panels) and ${\textsc {price}}$ charts (right panels) are almost identical to the ones from perceived riskiness (first bar, ‘risk’, in each group of bars).

When volatility and expected returns are the same but the charts are shown with different scaling (Condition ${\textsc {scale}}$; see second row of panels in Fig. 7), we find that between 49 and 72% of subjects (correctly) see no difference in profitability. However, up to 51% do see a difference. For the ${\textsc {positive stable}}$ and ${\textsc {increasing}}$ trends (first two groups of bars of each panel) the results are again similar to those for perceived riskiness—the asset shown with narrow scaling is perceived as the more profitable one as the (mostly positive) bars are displayed larger here. For the ${\textsc {negative stable}}$ and ${\textsc {decreasing}}$ trends, however, we find a marked difference: those 28% to 46% of subjects who do perceive a difference in profitability largely identify the asset displayed with wide scaling as more profitable—the mostly negative returns are shown with smaller bars and subjects are misled to think these are thus more profitable. Subjects fall into this ‘trap’ in between 18% and 33% of all decisions.

With regard to the change in scales between the two conditions, we observe similarly high numbers of ‘correct’ profitability assessments (in the sense of regarding the two essentially identical assets as equally profitable) for all trends except for ${\textsc {increasing}}$, for which we observe fewer mistakes in Condition ${\textsc {scale}}$. Overall, in 55% (${\textsc {same}}$) and 60% (${\textsc {scale}}$) of decisions, respectively, subjects assess the two as equally profitable.

*Result 7* Scaling can distort people’s perception about an asset’s profitability in pairwise comparisons of assets with different volatilities. With the same scale, more volatile assets of trends pos.stable and ${\textsc {increasing}}$ are regarded as more profitable; for trends neg.stable and ${\textsc {decreasing}}$, the opposite holds. With different scales, a large share perceives the low-volatility asset as more profitable.

*Support* The third row of panels in Fig. 7 presents Condition ${\textsc {volatility}}$, in which for ${\textsc {price}}$ charts there are more extreme prices for ${\textsc {high}}$-volatility assets—that is, the ${\textsc {high}}$-volatility asset yields higher prices with trends ${\textsc {positive stable}}$ and ${\textsc {increasing}}$ and lower prices with trends ${\textsc {negative stable}}$ and ${\textsc {decreasing}}$ (compared to the respective ${\textsc {low}}$-volatility assets). While almost 100% of subjects perceive the asset with the higher volatility as the riskier one with a ${\textsc {positive stable}}$ trend, we find 21% of subjects to assess the less risky asset as the more profitable one in this trend with ${\textsc {return}}$ charts. It seems that for profitability assessments subjects also take a lower volatility into account. Most notably, however, we observe the same pattern as above: for ${\textsc {positive stable}}$ and ${\textsc {increasing}}$ trends (first two groups of bars of each panel), most subjects perceive the more volatile asset as the more profitable one (as the returns bars are mostly positive, respectively the price mostly increases), while for the ${\textsc {negative stable}}$ and ${\textsc {decreasing}}$ trends (last two groups of bars of each panel), the opposite holds and the mostly negative returns/falling prices lead subjects to select the low-volatility asset as the more profitable one. For the latter two price trends the ‘indifferent’ choices are also markedly higher than for the positive price trends ($p<0.01$ for ${\textsc {return}}$ charts, $p<0.05$ for ${\textsc {price}}$ charts)—probably because subjects see both assets markedly going down and consider this a decision ‘between a rock and a hard place’, i.e. a choice between two equally bad alternatives.

Finally, for Condition ${\textsc {both}}$ we find shares of 29% to 66% of decisions in which subjects consider the asset with the lower volatility to be the more profitable one in the ${\textsc {negative stable}}$ and ${\textsc {decreasing}}$ trends. In addition, also in the two other trends (first two groups of bars) the share of subjects considering the low-volatility asset as the more profitable one is substantial, especially when ${\textsc {return}}$ charts are displayed. These shares of up to 38% are markedly higher than the respective shares for perceived riskiness ($p<0.01$).

Comparing the proportion of ‘correct’ assessments in the sense of regarding the asset with a higher average return as more profitable between conditions ${\textsc {volatility}}$ and ${\textsc {both}}$ we can again analyze the effect of adapting the scale with respect to the magnitude of returns. While on average the frequency of a ‘correct’ choice is higher (56%) with same scales (Condition ${\textsc {volatility}}$) compared to adapted scales (52%; Condition ${\textsc {both}}$), this difference is not statistically significant.

### Investment preferences in pairwise comparisons

In Task II we ask as a third question which of the two displayed assets subjects would rather invest in and incentivize this question by paying subjects one randomly determined return from the chosen asset. This allows us to examine the behavioral consequences of the scale effects reported above. The third bar in each set of bars in Fig. 7 (‘inv.’) shows the respective shares of investments in one of the two displayed assets.

In Condition ${\textsc {same}}$ a considerable fraction of subjects sees the two displayed assets as bearing different risks and profitabilities. A still higher share of between 33 and 86% of subjects decide to invest in either of the two. The share of ‘indifferent’ choices is smaller for investment preferences than for riskiness and profitability. This holds for each trend and both presentation formats. As the investment propensity is a function of both risk perception and return expectations, perceived differences in either of the two factors can lead to a difference in the investment propensity. Thus, the share of indifferent subjects should logically be smaller for investment propensity than it is for any of the two other variables. Additionally, the increase in decisiveness could also be attributable to the monetary incentives associated with this particular question.

For Condition ${\textsc {scale}}$, we find a considerable effect of the axis scale regarding investment decisions. Here, choices tend to be very similar to profitability assessments: as two identical assets are compared, assets presented on a ${\textsc {narrow}}$ scale are more frequently preferred for trends ${\textsc {positive stable}}$ and ${\textsc {increasing}}$, whereas for trends ${\textsc {negative stable}}$ and ${\textsc {decreasing}}$, the opposite holds (with the exception of ${\textsc {increasing}}$ in ${\textsc {price}}$ charts). Comparing conditions ${\textsc {same}}$ and ${\textsc {scale}}$ vertically reveals how the scale affects subjects’ behavior: we find on average a much less pronounced indifference between the two displayed assets in ${\textsc {same}}$ (44% vs. 57%, $p < 0.01$).

In the pairwise comparisons of conditions ${\textsc {volatility}}$ and ${\textsc {both}}$, we observe a number of diverging preferences—i.e., especially when return charts are displayed a large fraction chooses to invest in the ${\textsc {high}}$-volatility asset and a similarly large fraction chooses to invest in the ${\textsc {low}}$-volatility asset. The variation of the scale between these two conditions (same or different) does not result in a systematic difference in investment preferences.

Naturally, we are interested in how perceived riskiness and perceived profitability relate to people’s investment decisions. Comparing the shares of answers regarding investment preferences with the corresponding values concerning perceived riskiness (first bar, ‘risk’) and profitability (second bar, ‘profit.’) already hints at a meaningful relationship between these variables with the tendency of more investments in assets which are perceived as less risky and more profitable.

**Fig. 8:**
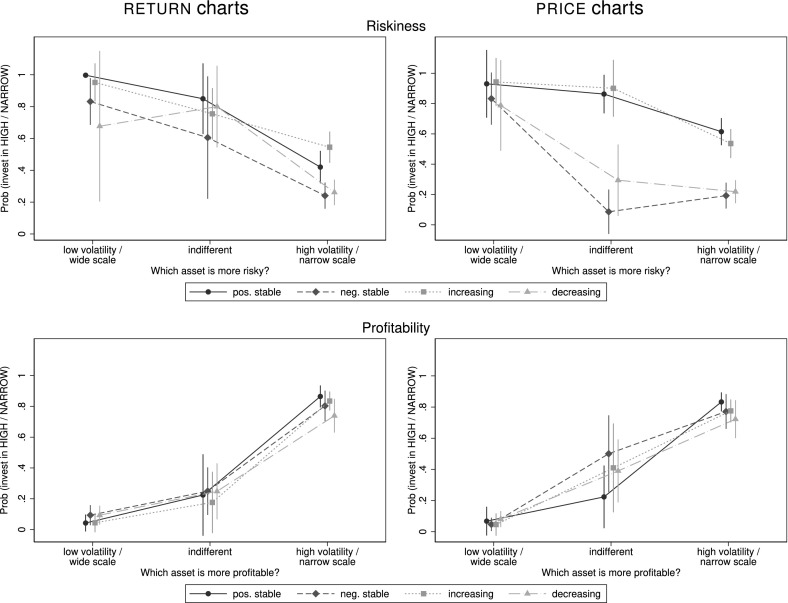
Predicted probabilities of investing in the ${\textsc {high}}$-volatility or ${\textsc {narrow}}$-scaled asset. This figure shows the predicted probabilities and 95%-confidence intervals of investing in the ${\textsc {high}}$-volatility (${\textsc {narrow}}$-scaled) asset depending on which asset is perceived as *more risky* (top) or as *more profitable* (bottom). Probabilities are estimated from a probit model with a dummy variable indicating whether the subject would invest in the ${\textsc {high}}$-volatility (${\textsc {narrow}}$-scaled) asset as the dependent variable and her choice regarding riskiness and profitability as independent variables. The numbers of observations for each estimation lie between 171 and 222 for different presentation formats and trends

For a more thorough analysis we estimate the probability with which a subject invests in either the ${\textsc {narrow}}$-scaled or in the ${\textsc {high}}$-volatility asset, respectively, depending on which asset she perceives as more risky and as more profitable, by running probit regressions.^19^ The resulting probabilities are plotted in Fig. 8.

*Result 8* Regarding one of two assets as more profitable and less risky leads to a higher probability of investing in this asset. Of the two factors profitability tends to be more important.

*Support* Regarding the effect of perceived riskiness (top panel of Fig. 8), we observe that in cases when a subject perceives the ${\textsc {low}}$ volatility or the ${\textsc {wide}}$-scaled asset as more risky, the probability that she invests in the ${\textsc {high}}$ volatility or ${\textsc {narrow}}$-scaled asset is between 68% and almost 100% across trends with return charts and even higher for price charts. Conversely, only around 25% tend to invest in this asset if it is perceived as riskier in trends ${\textsc {negative stable}}$ and ${\textsc {decreasing}}$, with a significantly higher number for trends ${\textsc {positive stable}}$ and in-creasing.

For price charts the probability of investing in the asset with higher perceived risk is 61 and 54%, respectively, with these trends—indicating that perceived risk is not necessarily the main determinant of investment behavior in the domain with mostly positive returns. Investing in the higher-volatility asset need not be a ‘wrong’ choice—especially in the case of a ${\textsc {negative stable}}$ trend having more volatile returns increases the chance that an investor could end up with a positive return. Such choices are thus in line with Prospect Theory (Kahneman and Tversky 1979) which postulates risk-seeking behavior in the loss domain, where returns of assets with a ${\textsc {negative stable}}$ trend mostly are (assuming zero return as subjects’ reference point). Hence, subjects who prefer the high-volatility asset over the low-volatility one in ${\textsc {negative stable}}$ (and with lower shares also in ${\textsc {increasing}}$ and ${\textsc {decreasing}}$ trends) should not be judged ‘irrational’ or ‘incorrect’, but can merely be risk-seeking in the loss domain.

Analyzing probabilities to invest depending on perceived profitability (bottom panel) we observe very similar estimates across all trends. If the ${\textsc {high}}$ volatility (${\textsc {narrow}}$-scaled) asset is perceived as more profitable, the probability of a subject investing in this asset is also very high (between 74 and 86%), and vice versa. As we observe comparable dynamics for all trends we conclude that perceived profitability is more important than perceived riskiness in these decisions. Subjects tend to invest in the asset which they regard as more profitable, even if they think it bears higher risk.

## Discussion and conclusion

In a novel experimental design we examined the impact of different vertical axis scales and presentation formats on risk perception, short- and long-term return expectations, and investment propensity. We explored return bar charts and price line charts for eight distinct assets, distinguished by either a low or a high volatility and one of four distinct return trends.

We found that varying the scale strongly affected people’s risk perception. Namely, a narrower scale of the vertical axis—that is, letting return bars and the line depicting the price, respectively, fill most of the available, vertical space in a chart—leads to significantly higher perceived riskiness of an asset. This result is robust to varying the chart’s presentation format (prices vs. returns) and the asset’s volatility and trend. Only when returns were consistently positive, risk perception was the same across different scalings.

Assets were usually perceived as riskier when returns were shown than when prices were shown. Regulations like the European standard for investor documents (Commission Regulation (EU) No 583/2010, 2010, p. 15) demand return bar charts and a vertical axis that *shall not compress the bars so as to make fluctuations in returns harder to distinguish*. We demonstrate that adapting the scale accordingly is reasonable with regard to recognizing yearly return variations *within* a single security, but at the same time makes it harder to identify differences *between* dissimilar securities.

We further reported that past returns predicted future return expectations almost perfectly, irrespective of the presentation format. Most subjects in our setting thus act as short-term trend-followers when predicting future prices and returns.

Risk perception is highly correlated with losses, which in turn drive investment behavior. This connects nicely to recent literature which also finds that risk perception is most strongly driven by ‘probability of loss’, and that this drives investment intentions (Anzoni and Zeisberger 2016) and prices (Huber et al. 2018). It is open to further investigation and beyond the scope of our paper, whether some of the scale effects we report would be mitigated or even increased if historical trends are reported visually alongside some quantitative measure of volatility (e.g., a stock’s beta or Value-at-Risk) or credit ratings by rating agencies.

Concerning investment choices, subjects tend to invest in the asset which they regard as more profitable even if they assess it to be riskier. Hence, in our setting perceived profitability was considered more important than perceived riskiness when making investment choices.

With regard to policy, our results have important implications: we already mentioned in the introduction the practical relevance in regulation. e.g. financial market regulators in the US require consumer information documents to contain return bar charts representing past performance, but do not require a standardized appearance.^20^ EU regulations also demand the presentation of return bar charts and, in addition, specific criteria regarding the presentation format. Yet, neither acknowledges the potentially distorting effects of the axis scale. In particular, the EU suggests *adapting the scale to the span of the bars* (Commission Regulation (EU) No 583/2010 2010, p. 15). As we have shown, this makes it harder to distinguish assets with different levels of volatility. hence, a well-meant regulatory rule might even have unintended negative consequences on investors’ decisions. An example is the case of two passive funds with the same tracking error but with different fee structures. As funds are required to report returns net of fees, this case essentially corresponds to a change of the trend of the data generating process. Many investors will not be able to detect the fund with the better fee structure if fund companies follow the current regulatory rules and adjust their scaling to the data. This also extends to the wider finance industry, especially the less-regulated and emerging parts like robo-advisers, online brokers, or financial websites (often featuring ads by all kinds of financial service providers), all of which provide and display financial information and charts in various ways.

To summarize, regulators, information providers, customers and consumers should be aware of—and attentive to—the potentially distorting effects of different axis scales in performance charts. While return bar charts seem appropriate, allowing issuers to adapt the axis scale arbitrarily leaves room for deliberate action aimed at distorting investors’ perceptions about risk. Keeping the presentation scale constant across different securities enables better identification of risk and therefore better comparisons and decisions.

## Electronic supplementary material

Below is the link to the electronic supplementary material.

Supplementary material 1 (pdf 812 KB)

## Footnotes

1 In developing the standardized KIID the European Commission employed a survey-based study by IFF Research, YouGov (2009). They mention different axis scales as a possible explanation for people’s difficulties in assessing past performance but do not attempt to offer any evidence confirming this intuition.

2 See Beattie and Jones (2008) for a survey of corporate reporting using graphs.

3 Violations of proportionality in representing the underlying data include, for example, omitting the zero line and using non-linear scales. See Tufte (1983) for a comprehensive exposition of visual data representations.

4 We elicit both risk perception and investment propensity on a 7-point Likert scale as in Anzoni and Zeisberger (2016). In eliciting expectations we ask for point estimates of future returns (prices) for both one and five year horizons (also see Weber et al. 2005; Glaser et al. 2018).

5
Glaser et al. (2007, 2018) discuss the impact of presenting returns vs. presenting prices and asking for returns vs. asking for prices. We consciously refrain from presenting one format and asking for the other, as the tasks we have seem demanding enough for subjects and we want to rule out potential confusion.

6 To avoid subjects being asked to compare two identical asset representations in setting ${\textsc {same}}$ (same scale and same volatility), we permute the returns of an asset in a way that preserves the asset’s characteristics but generates a marginally different path for comparison. One example can be seen in the first pair shown in Fig. E2 in Online Appendix E.

7 The return *R* of year $t \in [1,10]$ of the chosen asset of pair $p \in [1,8]$ in presentation format $F \in \{{\textsc {return}}, {\textsc {price}}\}$; if subjects are indifferent between two assets, either A or B is chosen randomly. For each of the two tasks they receive 5 euros $\times \, (1 + 2 \times R^F_{t,p})$.

8 As the probability of appearance of one particular chart is determined randomly for each subject, the number of observed decisions for each distinct combination of presentation format, volatility, and trend varies between 179 and 206.

9 To establish independence between observations we subtract the mean across all of a subject’s risk assessments from each data point. We then run non-parametric Fisher-Pitman permutation tests on these subject-demeaned data with 300,000 simulations as a more powerful alternative to Wilcoxon-Mann-Whitney tests (Kaiser 2007). If not stated otherwise, we use the same procedure throughout the paper. Wilcoxon sign-rank tests on the matched pairs of the original observations yield very similar results; multivariate regressions controlling for demographic variables also confirm this scale effect on risk perception, see Tables A2 and A3 in Online Appendix A.

10 Pearson’s correlation coefficients $\rho$ are between –0.10 and 0.14 across presentation formats and volatilities.

11 $p<0.01$ for 22 out of 24 pairwise comparisons between trends; for details see Online Appendix A.

12 This connects nicely to recent literature, e.g. by Anzoni and Zeisberger (2016) and Huber et al. (2018) showing that risk perception is mostly driven by losses. For further details see Online Appendix B.

13 As mentioned above, we consciously refrain from presenting one format and asking for the other—i.e., we ask for returns when presenting returns and ask for prices when presenting prices. For the analysis we only consider returns, either directly from subjects’ return estimates or calculated as the average annual difference between price estimates and the latest price.

14 With a Spearman’s rank correlation coefficient of –0.68 the relationships are far from perfect, though.

15 The average investment propensity for each asset and corresponding significance tests for differences between scales are provided in Table A7, the respective multivariate regressions controlling for demographic in Table A8, both in Online Appendix A.

16 The corresponding regression estimates as well as a more detailed analysis—also with respect to a potential scale effect—is provided in Section B.2 in Online Appendix B.

17 For comparisons between different choices and conditions in the pairwise comparisons of Task II we conduct two-sided Wald tests on the respective proportions and report the corresponding *p*-values in parentheses.

18 See Section B.3 in Online Appendix B for a more detailed analysis.

19 We run probit regressions with the pooled decisions across all conditions in which the two assets differ by at least one variable of interest—i.e., conditions ${\textsc {scale}}$, ${\textsc {volatility}}$, and ${\textsc {both}}$—and consider only those decisions in which a subject chose either one of the assets to invest in. Hence, the dependent variable is a binary variable taking the value 1 when a subject chose to invest in the high-volatility or narrow-scaled asset, respectively, and 0 otherwise. Her choices regarding riskiness and profitability act as explanatory variables. For estimating probabilities depending on the choice in riskiness, choices in profitability were assumed to be at their means and vice versa. We are aware that c.p. a higher volatility might have different effects than displaying the asset on a narrow scale; however, as we find very similar patterns within each condition, we present results from the pooled data only. The estimates for each individual condition are available upon request.

20 See Zimmer (2009) for US regulations regarding past performance information in prospectuses and Mercer et al. (2010) for mutual fund advertisements.
