# Supplementary material for: Scale matters: risk perception, return expectations, and investment propensity under different scalings
Source: Exp Econ. 2018 Dec 1;22(1):76–100. doi: 10.1007/s10683-018-09598-4 (PMC6373342; doi:10.1007/s10683-018-09598-4)
Supplement: Supplementary file 1 — Supplementary material 1 (pdf 812 KB) [file 10683_2018_9598_MOESM1_ESM.pdf]

Online Appendix to
'Scale matters: Risk perception, return expectations, and investment propensity under different scalings'

Christoph Huber and Jürgen Huber

A Additional Figures and Tables

Table A1: **Perceived risk of each hypothetical asset in return and price charts with wide and narrow scale.** This table shows the average perceived risk for each of the eight assets in RETURN charts and PRICE charts and each with a WIDE and a NARROW scale. The lines 'Diff.' show the average difference in perceived risk between presentations with scale NARROW and scale WIDE. *, **, and *** indicate the 10%, 5% and 1% significance levels of two-sided Fisher-Pitman permutation tests on the subject-demeaned data. The sample size N for each test is between 179 and 206.

	LOW volatility			
RETURN charts	POS. STABLE	NEG. STABLE	INCREASING	DECREASING
WIDE scale	1.93	5.70	3.83	4.96
NARROW scale	2.24	6.03	4.21	5.64
Diff.	0.31	0.33	0.38**	0.68***
PRICE charts				
WIDE scale	2.09	4.91	3.47	3.70
NARROW scale	2.08	5.45	4.05	5.29
Diff.	−0.01	0.54**	0.58***	1.59***
	HIGH volatility			
RETURN charts	POS. STABLE	NEG. STABLE	INCREASING	DECREASING
WIDE scale	3.43	5.54	4.05	5.39
NARROW scale	3.59	6.32	4.66	5.97
Diff.	0.16	0.78***	0.61***	0.58***
PRICE charts				
WIDE scale	2.96	5.20	4.07	5.04
NARROW scale	2.90	5.64	4.41	5.59
Diff.	−0.06	0.44**	0.34*	0.55***

Table A2: **Wilcoxon signed-rank tests for differences in perceived risk of each hypothetical asset in return and price charts between wide and narrow scales.** This table shows the average perceived risk for each of the eight assets in RETURN charts and PRICE charts and each with a WIDE and a NARROW scale for matched pairs. The lines 'Diff.' show the average difference in perceived risk between presentations with scale NARROW and scale WIDE. *, **, and *** indicate the 10%, 5% and 1% significance levels of two-sided matched-pairs Wilcoxon signed-rank tests. The sample size N for each test is between 36 and 50.

	LOW volatility			
RETURN charts	POS. STABLE	NEG. STABLE	INCREASING	DECREASING
WIDE scale	2.04	5.56	3.58	5.03
NARROW scale	2.43	5.92	4.23	5.67
Diff.	0.39*	0.35***	0.64***	0.64**
PRICE charts				
WIDE scale	2.12	4.83	3.22	3.52
NARROW scale	2.14	5.60	4.22	5.25
Diff.	0.02	0.77***	1.00***	1.73***

	HIGH volatility			
RETURN charts	POS. STABLE	NEG. STABLE	INCREASING	DECREASING
WIDE scale	3.15	5.52	4.18	5.40
NARROW scale	3.60	6.14	4.47	6.07
Diff.	0.45*	0.61**	0.29	0.67***
PRICE charts				
WIDE scale	2.79	4.96	3.70	4.95
NARROW scale	2.87	5.61	4.21	5.73
Diff.	0.09	0.65***	0.51*	0.78***

Table A3: **Fixed and random effects regressions for risk perception.** This table presents the estimated coefficients of generalized least squares regressions with risk perception (1 = "not risky at all" to 7 = "very risky") as the dependent variable. Scale = WIDE, Presentation format = PRICE, Volatility = HIGH, Trend = NEG. STABLE, Trend = INCREASING, Trend = DECREASING, Master's program, PhD program, Female, and Financial management are dummy variables taking the value 1 if the respective condition is fulfilled. Master's program and PhD program refer to the course of study a subject is currently enrolled in; Financial management indicates whether a subject has completed the introductory course.

Dependent variable: *Risk perception*	Fixed effects (1)	Random effects (2)
Scale = WIDE	−0.480***	−0.478***
	(0.051)	(0.050)
Presentation format = PRICE	−0.403***	−0.403***
	(0.050)	(0.050)
Volatility = HIGH	0.571***	0.570***
	(0.051)	(0.050)
Trend = NEG. STABLE	3.010***	2.993***
	(0.071)	(0.071)
Trend = INCREASING	1.490***	1.476***
	(0.072)	(0.071)
Trend = DECREASING	2.596***	2.581***
	(0.072)	(0.071)
Master's program		0.002
		(0.090)
PhD program		0.437
		(0.564)
Study semester		−0.011
		(0.017)
Age		0.006
		(0.020)
Female		−0.136
		(0.091)
Financial management		−0.019
		(0.093)
Math grade		−0.018
		(0.041)
Risk attitude (general)		−0.022
		(0.034)
Risk attitude (financial)		0.002
		(0.036)
Constant	2.769***	2.907***
	(0.066)	(0.449)
Fixed effects	Yes	No
Observations	3,088	3,088
Number of subjects	193	193
R2 within	0.458	0.458
R2 between	0.057	0.076
R2 overall	0.423	0.425

Table A4: **Average one-year return forecasts of each hypothetical asset in return and price charts with wide and narrow scale.** This table shows the average one-year return forecasts for each of the eight assets in RETURN charts and PRICE charts and each with a WIDE and a NARROW scale. The lines 'Diff.' show the average difference in one-year return forecasts between presentations with scale NARROW and scale WIDE. *, **, and *** indicate the 10%, 5% and 1% significance levels of two-sided Fisher-Pitman permutation tests on the subject-demeaned data. The sample size N for each test is between 179 and 206.

	LOW volatility			
RETURN charts	POS. STABLE	NEG. STABLE	INCREASING	DECREASING
WIDE scale	3.62	−2.60	2.89	−1.67
NARROW scale	4.02	−2.99	3.13	−1.90
Diff.	0.40	−0.39	0.24	−0.23
PRICE charts				
WIDE scale	3.32	−4.34	3.27	−2.86
NARROW scale	2.33	−6.90	2.27	−2.31
Diff.	−0.98	−2.56**	−1.00*	0.55

	HIGH volatility			
RETURN charts	POS. STABLE	NEG. STABLE	INCREASING	DECREASING
WIDE scale	5.04	−2.78	3.88	−1.08
NARROW scale	4.57	−2.27	3.74	−0.74
Diff.	−0.47	0.51	−0.14	0.35
PRICE charts				
WIDE scale	−0.50	−2.67	2.22	−0.77
NARROW scale	2.87	−5.97	1.54	−3.05
Diff.	3.37**	−3.30	−0.68	−2.27

Table A5: **Average five-year return forecasts of each hypothetical asset in return and price charts with wide and narrow scale.** This table shows the average five-year return forecasts for each of the eight assets in RETURN charts and PRICE charts and each with a WIDE and a NARROW scale. The lines 'Diff.' show the average difference in five-year return forecasts between presentations with scale NARROW and scale WIDE. *, **, and *** indicate the 10%, 5% and 1% significance levels of two-sided Fisher-Pitman permutation tests on the subject-demeaned data. The sample size N for each test is between 179 and 206.

	LOW volatility			
	Asset 1	Asset 2	Asset 3	Asset 4
RETURN charts	POS. STABLE	NEG. STABLE	INCREASING	DECREASING
WIDE scale	3.95	−2.87	2.57	−0.40
NARROW scale	4.79	−3.17	2.10	−0.94
Diff.	0.85*	−0.30	−0.46	−0.54
PRICE charts				
WIDE scale	2.51	−4.92	1.18	−0.17
NARROW scale	2.28	−6.21	0.15	−1.00
Diff.	−0.23	−1.29	−1.03**	−0.83

	HIGH volatility			
	Asset 5	Asset 6	Asset 7	Asset 8
RETURN charts	POS. STABLE	NEG. STABLE	INCREASING	DECREASING
WIDE scale	6.47	−4.71	4.89	−2.25
NARROW scale	6.27	−4.66	4.41	−1.69
Diff.	−0.20	0.05	−0.48	0.56
PRICE charts				
WIDE scale	1.09	−3.65	0.07	−0.34
NARROW scale	1.58	−3.39	0.49	−1.61
Diff.	0.49	0.27	0.42	−1.28

Table A6: **Fixed and random effects regressions for expected returns.** This table presents the estimated coefficients of generalized least squares regressions with expected returns as the dependent variable. Scale = WIDE, Presentation format = PRICE, Volatility = HIGH, Trend = NEG. STABLE, Trend = INCREASING, Trend = DECREASING, Master's program, PhD program, Female, and Financial management are dummy variables taking the value 1 if the respective condition is fulfilled. Master's program and PhD program refer to the course of study a subject is currently enrolled in; Financial management indicates whether a subject has completed the introductory course.

Dependent variable:	*1-year expected return*		*5-year expected return*	
	Fixed effects	Random effects	Fixed effects	Random effects
	(1)	(2)	(3)	(4)
Scale = WIDE	1.079	0.998	0.142	0.186
	(0.770)	(0.759)	(0.234)	(0.234)
Presentation format = PRICE	−1.195	−1.196	−2.077***	−2.071***
	(0.757)	(0.757)	(0.230)	(0.233)
Volatility = HIGH	0.773	1.020	−0.169	−0.100
	(0.770)	(0.760)	(0.235)	(0.234)
Trend = NEG. STABLE	−8.269***	−8.387***	−8.271***	−8.259***
	(1.081)	(1.067)	(0.329)	(0.329)
Trend = INCREASING	−1.199	−1.576	−1.586***	−1.599***
	(1.091)	(1.070)	(0.332)	(0.330)
Trend = DECREASING	−6.057***	−6.440***	−4.823***	−4.873***
	(1.094)	(1.077)	(0.333)	(0.332)
Master's program		1.170		−0.055
		(0.919)		(0.315)
PhD program		1.675		0.709
		(5.783)		(1.986)
Study semester		−0.612***		−0.061
		(0.176)		(0.061)
Age		0.414**		−0.020
		(0.210)		(0.072)
Female		−0.039		−0.041
		(0.937)		(0.322)
Financial management		1.850**		0.655**
		(0.958)		(0.329)
Math grade		0.185		0.257*
		(0.420)		(0.144)
Risk attitude (general)		0.059		0.104
		(0.351)		(0.121)
Risk attitude (financial)		0.219		−0.129
		(0.369)		(0.127)
Constant	3.908***	−4.832	4.606***	4.508***
	(1.007)	(4.651)	(0.306)	(1.591)
Fixed effects	Yes	No	Yes	No
Observations	3,088	3,088	3,087	3,087
Number of subjects	193	193	193	193
R2 within	0.028	0.028	0.221	0.221
R2 between	0.029	0.106	0.075	0.104
R2 overall	0.028	0.034	0.207	0.210

Table A7: **Average propensities to invest for each hypothetical asset in return and price charts with wide and narrow scale.** This table shows the average likelihood to invest for each of the eight assets in RETURN charts and PRICE charts and each with a WIDE and a NARROW scale. The lines 'Diff.' show the average difference in likelihood to invest between presentations with scale NARROW and scale WIDE. *, **, and *** indicate the 10%, 5% and 1% significance levels of two-sided Fisher-Pitman permutation tests on the subject-demeaned data. The sample size N for each test is between 179 and 206.

	LOW volatility			
RETURN charts	POS. STABLE	NEG. STABLE	INCREASING	DECREASING
WIDE scale	5.96	1.41	4.16	2.28
NARROW scale	6.09	1.38	4.32	1.99
Diff.	0.13	−0.02	0.16	−0.29
PRICE charts				
WIDE scale	5.85	1.81	4.41	3.23
NARROW scale	5.66	1.87	4.33	2.69
Diff.	−0.19	0.06	−0.08	−0.55***

	HIGH volatility			
RETURN charts	POS. STABLE	NEG. STABLE	INCREASING	DECREASING
WIDE scale	5.17	1.83	4.38	2.11
NARROW scale	5.22	1.72	4.30	2.15
Diff.	0.05	−0.11	−0.08	0.04
PRICE charts				
WIDE scale	5.43	2.24	4.68	2.81
NARROW scale	5.33	2.41	4.69	2.43
Diff.	−0.10	0.17	0.01	−0.39**

Table A8: **Fixed and random effects regressions for investment propensity.** This table presents the estimated coefficients of generalized least squares regressions with investment propensity (1 = "not likely at all" to 7 = "very likely") as the dependent variable. Scale = WIDE, Presentation format = PRICE, Volatility = HIGH, Trend = NEG. STABLE, Trend = INCREASING, Trend = DECREASING, Master's program, PhD program, Female, and Financial management are dummy variables taking the value 1 if the respective condition is fulfilled. Master's program and PhD program refer to the course of study a subject is currently enrolled in; Financial management indicates whether a subject has completed the introductory course.

Dependent variable: *Investment propensity*	Fixed effects (1)	Random effects (2)
Scale = WIDE	0.055	0.059
	(0.047)	(0.047)
Presentation format = PRICE	0.326***	0.326***
	(0.047)	(0.047)
Volatility = HIGH	−0.061	−0.052
	(0.047)	(0.047)
Trend = NEG. STABLE	−3.807***	−3.791***
	(0.066)	(0.066)
Trend = INCREASING	−1.221***	−1.207***
	(0.067)	(0.066)
Trend = DECREASING	−3.186***	−3.164***
	(0.067)	(0.067)
Master's program		−0.091
		(0.078)
PhD program		−0.143
		(0.492)
Study semester		0.010
		(0.015)
Age		−0.007
		(0.018)
Female		0.022
		(0.080)
Financial management		0.065
		(0.082)
Math grade		−0.031
		(0.036)
Risk attitude (general)		0.010
		(0.030)
Risk attitude (financial)		0.058*
		(0.031)
Constant	5.463***	5.385***
	(0.062)	(0.392)
Fixed effects	Yes	No
Observations	3,088	3,088
Number of subjects	193	193
R2 within	0.594	0.594
R2 between	0.142	0.174
R2 overall	0.563	0.566

Table A9: **Differences in perceived risk between trends.** This table shows the differences in perceived risk between trends for LOW- and HIGH-volatility assets in RETURN and PRICE charts, pooled across scales. *, **, and *** indicate the 10%, 5% and 1% significance levels of two-sided Fisher-Pitman permutation tests on the subject-demeaned data.

	RETURN charts					
	LOW volatility			HIGH volatility		
	POS. STABLE	NEG. STABLE	INCR.	POS. STABLE	NEG. STABLE	INCR.
NEG. STABLE	−3.78***			−2.43***		
INCREASING	−1.92***	1.86***		−0.85***	1.58***	
DECREASING	−3.23***	0.55***	−1.31***	−2.18***	0.25*	−1.33***

	PRICE charts					
	LOW volatility			HIGH volatility		
	POS. STABLE	NEG. STABLE	INCR.	POS. STABLE	NEG. STABLE	INCR.
NEG. STABLE	−3.09***			−2.50***		
INCREASING	−1.68***	1.41***		−1.30***	1.20***	
DECREASING	−2.44***	0.65***	−0.76***	−2.38***	0.12	−1.07***

Table A10: **Differences in investment propensity between trends.** This table shows the differences in investment propensity between trends for LOW- and HIGH-volatility assets in RETURN and PRICE charts, pooled across scales. *, **, and *** indicate the 10%, 5% and 1% significance levels of two-sided Fisher-Pitman permutation tests on the subject-demeaned data.

	RETURN charts					
	LOW volatility			HIGH volatility		
	POS. STABLE	NEG. STABLE	INCR.	POS. STABLE	NEG. STABLE	INCR.
NEG. STABLE	4.63***			3.42***		
INCREASING	1.80***	−2.83***		0.86***	−2.56***	
DECREASING	3.91***	−0.73***	2.11***	3.07***	−0.36**	2.21***

	PRICE charts					
	LOW volatility			HIGH volatility		
	POS. STABLE	NEG. STABLE	INCR.	POS. STABLE	NEG. STABLE	INCR.
NEG. STABLE	3.92***			3.05***		
INCREASING	1.38***	−2.54***		0.69***	−2.36***	
DECREASING	2.80***	−1.12***	1.42***	2.76***	−0.29*	2.07***

Table A11: **Differences in investment propensity between scales.** This table shows Wald tests for differences in regression coefficients between NARROW and WIDE scales for the models with RETURN (4a) and PRICE (4b) charts from Table 2 in the main text. *, **, and *** indicate the 10%, 5% and 1% significance levels.

	RETURN charts			
LOW volatility	POS. STABLE	NEG. STABLE	INCREASING	DECREASING
F-statistic	0.18	0.00	0.79	1.56
HIGH volatility	POS. STABLE	NEG. STABLE	INCREASING	DECREASING
F-statistic	0.35	0.34	0.07	0.00
	PRICE charts			
LOW volatility	POS. STABLE	NEG. STABLE	INCREASING	DECREASING
F-statistic	0.92	0.44	0.06	7.79***
HIGH volatility	POS. STABLE	NEG. STABLE	INCREASING	DECREASING
F-statistic	0.24	1.21	0.00	3.52*

Table A12: **Descriptive statistics of the main dependent variables.** This table shows the median, the mean, and the standard deviation for each of the main outcome variables *risk perception*, (1-year and 5-year avg. yearly) *expected return*, and *investment propensity.* The sample sizes are $N = 3087$ for 5-year avg. yearly expected returns and $N = 3088$ for each of the other three variables.

	Median	Mean	Std. Deviation
Risk perception	5.00	4.38	1.90
1-year expected return	1.00	0.36	21.44
5-year avg. yearly expected return	0.83	−0.11	7.38
Investment propensity	3.00	3.58	2.03

Table A13: **Demographic composition for return charts.** This table summarizes the demographic composition for each RETURN chart consisting of a distinct combination of volatility, trend, and scale. The last column (p-values) represents p-values of Kruskal-Wallis equality-of-populations rank tests between all possible combinations with RETURN charts and either LOW volatility (upper panel) or HIGH volatility (lower panel).

RETURN charts

		LOW volatility								
	Trend	POS. STABLE		NEG. STABLE		INCREASING		DECREASING		
Variable	Scale	narrow	wide	narrow	wide	narrow	wide	narrow	wide	p-value
Course of study (%)	Bachelor's	45.87	58.51	48.08	48.98	54.35	53.40	52.08	54.22	0.66
	Master's	53.21	41.49	50.96	51.02	45.65	46.60	47.92	45.78	
	PhD	0.92	0.00	0.96	0.00	0.00	0.00	0.00	0.00	
Semester	Median	5	4	5	6	5	5	5.5	5	0.94
	Mean	5.79	5.38	5.74	6.03	5.55	5.80	5.89	5.46	
	S.D.	3.36	3.22	3.08	3.78	2.98	3.38	3.26	3.20	
Age	Median	23	22	23	23	23	23	23	23	0.50
	Mean	23.09	22.35	23.03	23.20	22.84	23.11	22.78	22.75	
	S.D.	2.46	2.27	2.77	2.89	2.46	2.92	2.38	2.33	
Female (%)		0.41	0.53	0.50	0.48	0.41	0.50	0.51	0.40	0.42
Financial management (%)		0.50	0.46	0.50	0.54	0.54	0.53	0.46	0.51	0.87
Math grade (%)	1	21.10	24.47	25.00	24.49	26.09	19.42	26.04	24.10	0.76
	2	38.53	39.36	43.27	34.69	33.70	37.86	35.42	28.92	
	3	26.61	22.34	24.04	26.53	25.00	27.18	25.00	32.53	
	4	12.84	10.64	5.77	11.22	13.04	13.59	12.50	12.05	
	5	0.92	3.19	1.92	3.06	2.17	1.94	1.04	2.41	
Risk attitude (general)	Median	4	4	4	4	4	4	4	4	0.85
	Mean	3.83	3.97	3.77	3.90	3.99	3.99	3.83	4.05	
	S.D.	1.41	1.48	1.41	1.45	1.34	1.36	1.53	1.37	
Risk attitude (financial)	Median	3	3	3	3	4	3	3	3	0.84
	Mean	3.48	3.28	3.38	3.43	3.53	3.46	3.28	3.47	
	S.D.	1.49	1.46	1.29	1.47	1.35	1.41	1.45	1.40	
N		109	94	104	98	92	103	96	83	

		HIGH volatility								
	Trend	POS. STABLE		NEG. STABLE		INCREASING		DECREASING		
Variable	Scale	narrow	wide	narrow	wide	narrow	wide	narrow	wide	p-value
Course of study (%)	Bachelor's	50.53	46.59	47.00	51.61	51.00	56.99	51.96	51.06	0.91
	Master's	48.42	53.41	52.00	47.31	48.00	43.01	47.06	47.87	
	PhD	1.05	0.00	1.00	1.08	1.00	0.00	0.98	1.07	
Semester	Median	6	4	6	6	6	4	4	5	0.63
	Mean	5.95	5.42	6.27	6.08	5.94	5.47	5.76	5.62	
	S.D.	3.29	3.25	3.62	3.57	3.39	3.39	3.57	3.50	
Age	Median	23	23	23	23	23	22	23	23	0.89
	Mean	23.05	22.93	23.32	22.94	22.89	22.75	22.99	23.13	
	S.D.	2.84	2.65	2.73	2.57	2.43	2.71	2.95	2.89	
Female (%)		0.41	0.40	0.43	0.46	0.44	0.45	0.46	0.40	0.97
Financial management (%)		0.47	0.55	0.51	0.57	0.52	0.47	0.48	0.52	0.95
Math grade (%)	1	22.11	20.45	21.00	23.66	25.00	27.96	21.57	21.28	0.86
	2	40.00	40.91	41.00	40.86	34.00	38.71	40.20	36.17	
	3	26.32	28.41	23.00	22.58	28.00	19.35	22.55	27.66	
	4	9.47	9.09	13.00	10.75	10.00	12.90	12.75	12.77	
	5	2.11	1.14	2.00	2.15	3.00	1.08	2.94	2.13	
Risk attitude (general)	Median	4	4	4	4	4	4	4	4	0.97
	Mean	3.95	3.97	4.10	4.01	3.95	3.87	4.02	3.94	
	S.D.	1.34	1.46	1.34	1.25	1.49	1.38	1.36	1.35	
Risk attitude (financial)	Median	3	4	3	4	3	3	3	3	0.93
	Mean	3.33	3.56	3.46	3.54	3.40	3.35	3.40	3.48	
	S.D.	1.33	1.45	1.37	1.40	1.42	1.42	1.34	1.43	
N		95	88	100	93	100	93	102	94	

Table A14: **Demographic composition for price charts.** This table summarizes the demographic composition for each PRICE chart consisting of a distinct combination of volatility, trend, and scale. The last column (p-values) represents p-values of Kruskal-Wallis equality-of-populations rank tests between all possible combinations with PRICE charts and either LOW volatility (upper panel) or HIGH volatility (lower panel).

PRICE charts										
		LOW volatility								
	Trend	POS. STABLE		NEG. STABLE		INCREASING		DECREASING		
Variable	Scale	narrow	wide	narrow	wide	narrow	wide	narrow	wide	p-value
Course of study (%)	Bachelor's	49.46	57.14	57.73	43.69	44.44	47.47	50.00	47.78	0.38
	Master's	49.46	42.86	41.24	55.34	55.56	52.53	48.96	51.11	
	PhD	1.08	0.00	1.03	0.97	0.00	0.00	1.04	1.11	
Semester	Median	5	4	4	6	4	5	6	5	0.62
	Mean	5.83	5.26	5.33	6.04	5.48	5.74	6.14	5.89	
	S.D.	3.44	3.11	3.04	3.59	3.13	3.35	3.51	3.50	
Age	Median	23	22	22	23	23	23	23	22	0.59
	Mean	22.89	22.60	22.93	23.29	22.81	22.87	23.25	22.82	
	S.D.	2.50	2.75	2.50	2.91	2.34	2.51	2.82	2.85	
Female (%)		0.46	0.48	0.46	0.48	0.45	0.47	0.45	0.50	1.00
Financial management (%)		0.53	0.48	0.48	0.50	0.53	0.49	0.48	0.51	0.99
Math grade (%)	1	15.05	20.41	22.68	25.24	17.17	18.18	26.04	24.44	0.89
	2	40.86	39.80	34.02	35.92	36.36	44.44	34.38	36.67	
	3	34.41	24.49	29.90	22.33	32.32	22.22	25.00	27.78	
	4	8.60	12.24	13.40	12.62	10.10	13.13	14.58	8.89	
	5	1.08	3.06	0.00	3.88	4.04	2.02	0.00	2.22	
Risk attitude (general)	Median	4	4	4	4	4	4	4	4	0.73
	Mean	3.89	3.97	4.03	3.94	4.12	3.88	3.91	3.74	
	S.D.	1.48	1.42	1.40	1.42	1.48	1.33	1.35	1.30	
Risk attitude (financial)	Median	3	3	3	3	4	3	3	3	0.87
	Mean	3.45	3.31	3.37	3.44	3.56	3.29	3.41	3.21	
	S.D.	1.40	1.41	1.41	1.44	1.52	1.33	1.36	1.29	
N		93	98	97	103	99	99	96	90	

		HIGH volatility								
	Trend	POS. STABLE		NEG. STABLE		INCREASING		DECREASING		
Variable	Scale	narrow	wide	narrow	wide	narrow	wide	narrow	wide	p-value
Course of study (%)	Bachelor's	52.43	48.54	56.00	52.87	57.14	53.68	53.19	50	0.94
	Master's	47.57	50.49	44.00	47.13	42.86	45.26	45.74	50	
	PhD	0.00	0.97	0.00	0.00	0.00	1.05	1.06	0	
Semester	Median	5	5	6	5	5	4	5	6	0.81
	Mean	5.55	5.82	6.26	5.94	5.49	5.58	6.02	5.92	
	S.D.	3.13	3.60	3.57	3.50	3.05	3.46	3.19	3.66	
Age	Median	23	23	23	23	23	22	23	23	0.82
	Mean	22.91	23.02	23.19	23.02	22.73	22.64	23.04	23.20	
	S.D.	2.70	2.44	2.68	2.63	2.64	2.53	2.77	2.85	
Female (%)		0.46	0.43	0.53	0.37	0.46	0.38	0.43	0.40	0.37
Financial management (%)		0.54	0.50	0.53	0.52	0.48	0.47	0.56	0.50	0.93
Math grade (%)	1	23.30	23.30	23.00	27.59	27.47	29.47	27.66	22.92	0.80
	2	39.81	38.83	39.00	39.08	35.16	30.53	43.62	36.46	
	3	28.16	25.24	23.00	22.99	20.88	26.32	19.15	21.88	
	4	7.77	8.74	14.00	9.20	14.29	10.53	7.45	16.67	
	5	0.97	3.88	1.00	1.15	2.20	3.16	2.13	2.08	
Risk attitude (general)	Median	4	4	4	4	4	4	4	4	0.97
	Mean	3.83	4.04	3.89	4.03	3.88	3.97	3.97	3.98	
	S.D.	1.47	1.29	1.49	1.34	1.24	1.41	1.46	1.43	
Risk attitude (financial)	Median	3	4	3	4	3	3	3	4	0.78
	Mean	3.36	3.57	3.44	3.55	3.34	3.44	3.41	3.64	
	S.D.	1.44	1.32	1.51	1.43	1.34	1.36	1.46	1.43	
N		103	103	100	87	91	95	94	96	

Table A15: **Number of observations in Task I.**

Decision	Presentation format	Volatility	Trend	Scale	Observations
1	RETURN	LOW	POS. STABLE	NARROW	109
2	RETURN	LOW	POS. STABLE	WIDE	94
3	RETURN	LOW	INCREASING	NARROW	104
4	RETURN	LOW	INCREASING	WIDE	98
5	RETURN	LOW	NEG. STABLE	NARROW	92
6	RETURN	LOW	NEG. STABLE	WIDE	103
7	RETURN	LOW	DECREASING	NARROW	96
8	RETURN	LOW	DECREASING	WIDE	83
9	RETURN	HIGH	POS. STABLE	NARROW	95
10	RETURN	HIGH	POS. STABLE	WIDE	88
11	RETURN	HIGH	INCREASING	NARROW	100
12	RETURN	HIGH	INCREASING	WIDE	93
13	RETURN	HIGH	NEG. STABLE	NARROW	100
14	RETURN	HIGH	NEG. STABLE	WIDE	93
15	RETURN	HIGH	DECREASING	NARROW	102
16	RETURN	HIGH	DECREASING	WIDE	94
17	PRICE	LOW	POS. STABLE	NARROW	93
18	PRICE	LOW	POS. STABLE	WIDE	98
19	PRICE	LOW	INCREASING	NARROW	97
20	PRICE	LOW	INCREASING	WIDE	103
21	PRICE	LOW	NEG. STABLE	NARROW	99
22	PRICE	LOW	NEG. STABLE	WIDE	99
23	PRICE	LOW	DECREASING	NARROW	96
24	PRICE	LOW	DECREASING	WIDE	90
25	PRICE	HIGH	POS. STABLE	NARROW	103
26	PRICE	HIGH	POS. STABLE	WIDE	103
27	PRICE	HIGH	INCREASING	NARROW	100
28	PRICE	HIGH	INCREASING	WIDE	87
29	PRICE	HIGH	NEG. STABLE	NARROW	91
30	PRICE	HIGH	NEG. STABLE	WIDE	95
31	PRICE	HIGH	DECREASING	NARROW	94
32	PRICE	HIGH	DECREASING	WIDE	96

Table A16: **Number of observations in Task II.**

Decision	Presentation format	Condition	Trend	Observations
1	RETURN	SAME	POS. STABLE	91
2	RETURN	SAME	INCREASING	93
3	RETURN	SAME	NEG. STABLE	99
4	RETURN	SAME	DECREASING	103
5	RETURN	SCALE	POS. STABLE	86
6	RETURN	SCALE	INCREASING	95
7	RETURN	SCALE	NEG. STABLE	101
8	RETURN	SCALE	DECREASING	104
9	RETURN	VOLATILITY	POS. STABLE	94
10	RETURN	VOLATILITY	INCREASING	93
11	RETURN	VOLATILITY	NEG. STABLE	103
12	RETURN	VOLATILITY	DECREASING	96
13	RETURN	BOTH	POS. STABLE	90
14	RETURN	BOTH	INCREASING	93
15	RETURN	BOTH	NEG. STABLE	105
16	RETURN	BOTH	DECREASING	98
17	PRICE	SAME	POS. STABLE	75
18	PRICE	SAME	INCREASING	86
19	PRICE	SAME	NEG. STABLE	92
20	PRICE	SAME	DECREASING	85
21	PRICE	SCALE	POS. STABLE	89
22	PRICE	SCALE	INCREASING	84
23	PRICE	SCALE	NEG. STABLE	74
24	PRICE	SCALE	DECREASING	91
25	PRICE	VOLATILITY	POS. STABLE	89
26	PRICE	VOLATILITY	INCREASING	76
27	PRICE	VOLATILITY	NEG. STABLE	75
28	PRICE	VOLATILITY	DECREASING	74
29	PRICE	BOTH	POS. STABLE	85
30	PRICE	BOTH	INCREASING	96
31	PRICE	BOTH	NEG. STABLE	84
32	PRICE	BOTH	DECREASING	73

B Further analyses

B.1 Potential asset- and chart-specific drivers of risk perception

As we mention in the main text, people perceive risk as significantly higher when presented with RETURN charts as compared to PRICE charts (all differences have the same sign; five out of eight p-values are significant at $p < 0.01$, see Table B1). The observed effects of either a NARROW or a WIDE scale reported above are robust to the presentation format and take comparable magnitudes in both, RETURN and PRICE charts, but there is a level effect, with risk usually being perceived as significantly higher when the presentation format is RETURN charts. While most stock information systems usually present price charts, the EU demands that the KIID contains return charts of a fund's preceding ten years. If the regulator's intention is to ensure that investors act carefully, this approach seems sensible and likely to achieve the intended goal. However, while return charts tend to elevate perceived risk, we cannot tell whether people's risk assessments become more accurate as a result.

Table B1: **Risk perception in return and price charts.** This table shows the perceived risk for each of the eight assets in RETURN and PRICE charts, pooled across scales. The lines 'Diff.' show the average differences. *, **, and *** indicate the 10%, 5% and 1% significance levels of two-sided Fisher-Pitman permutation tests on the subject-demeaned data.

	LOW volatility			
	POS. STABLE	NEG. STABLE	INCREASING	DECREASING
RETURN	2.09	5.87	4.01	5.32
PRICE	2.08	5.17	3.76	4.52
Diff.	0.01	0.70***	0.25	0.80***

	HIGH volatility			
	POS. STABLE	NEG. STABLE	INCREASING	DECREASING
RETURN	3.51	5.94	4.37	5.69
PRICE	2.93	5.43	4.24	5.31
Diff.	0.58***	0.51***	0.13	0.38***

Our data also allows us to contribute to the ongoing discussion on which factors drive risk perception. Previous research has found that asset characteristics other than historical or theoretical standard deviation drive risk perception (e.g. Unser, 2000; Veld and Veld-Merkoulova, 2008; Anzoni and Zeisberger, 2016). We consider the relationship between possible alternative determinants of risk and subjects' perceived risk. Fig. B1 plots average perceived risk as a function of historical standard deviation, average historical return, maximum loss, last return, expected value of loss, and loss probability. One can easily see that standard deviation is indeed unable to sufficiently explain the risk subjects perceive. In contrast, we observe strong correlations between perceived risk and asset characteristics associated with returns,

in particular with losses.[1] This is in line with recent literature (e.g. Anzoni and Zeisberger, 2016; Huber et al., 2018) who report that probability of loss is the main driver of perceived risk. With an R^2 of 0.84 the loss probability also has the highest explanatory power of the factors we consider in Fig. B1. Note that this finding is at odds with most of the neoclassical finance literature which uses standard deviation of returns as the main risk measure.

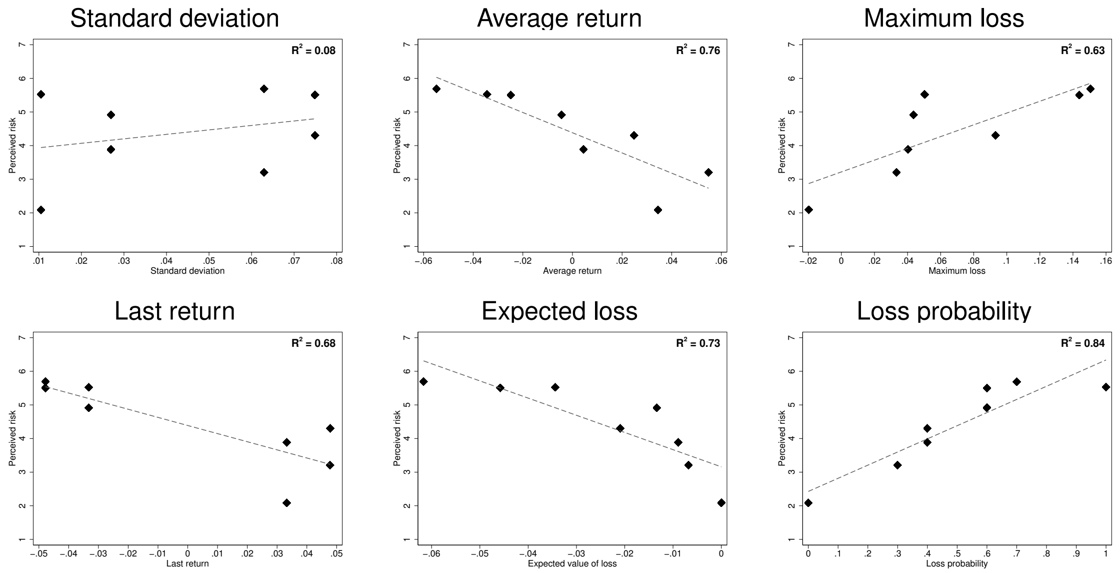

Figure B1: **Some potential determinants of perceived risk.** This figure shows scatter plots for the average perceived risk (depicted on the vertical axis) observed as a function of potential determinants associated with risk perception (depicted on the horizontal axis) and corresponding R^2 on the top right of each panel. Potential risk drivers analyzed are standard deviation, average return, maximum loss, last return, expected loss, and loss probability.

B.2 Investment preferences in individual assessments

In an attempt to explain investment behavior more generally, Nosić and Weber (2010) and Kaufmann et al. (2013) point out that in a behavioral risk-return framework risk taking – and therefore being willing to invest in risky assets – is driven not just by the historical return and volatility of an asset, but by the investor's risk attitude, her risk perception, and her subjective return expectation regarding the asset: thus, *Risk Taking* = f(*Perceived Return; Risk Attitude; Perceived Risk*) (also see Sarin and Weber, 1993; Jia et al., 1999). We run least squares regressions similar to Nosić and Weber (2010) to examine subjects' investment behavior. Participants' risk attitudes are captured by subject-fixed effects. Detailed results are provided in Table B2.

[1] Note that – given our experimental set-up – these characteristics are highly correlated. Therefore, we are unable to identify which of these characteristics is the driving force behind perceived risk.

The first regression shows that higher historical return and lower historical volatility of the asset increase subjects' propensity to invest (measured on a scale from 1 to 7). Model (2) regresses investment propensity on subjective measures of risk and return – that is, subjects' perceptions. The estimates suggest that lower (subjective) perceived risk of an asset (given a specific presentation format) and higher subjective long-term expected returns increase the likelihood of investing. Combining (1) and (2) in Model (3) only marginally increases the model's explanatory power compared to the one with only subjective regressors, confirming the intuition put forward above: investment propensity predominantly relies on people's subjective perceptions.

In Section 3.1 in the main text, we have demonstrated that the scale in which an asset's performance is presented drives people's perceptions about its risk. Therefore, we estimate an additional model, substituting risk perception by the interaction of the asset and the chart's scale; for the full data set in Model (4) as well as for return charts in Model (4a) and for price charts in Model (4b).[2] While the coefficients remain comparable in magnitude and significance, we can now run Wald tests for differences between scales for each of the eight assets. In the full-data Model (4), we find significantly lower investment propensity for the low-volatility asset with trend DECREASING when presented with a NARROW scale ($p < 0.01$). For five out of seven other assets, people's willingness to invest is also lower with a NARROW scale but the differences are insignificant.

B.3 Differences between conditions in pairwise comparisons

In the main text we focus our discussion of the results from pairwise comparisons on the scale effect in each of the four conditions. Additionally, when we compare the *perceived riskiness* assessments in Fig. B2 vertically, i.e. comparing the same price trends across the four different conditions (SAME, SCALE, VOLATILITY, and BOTH), the upper two (with equal volatility) and the lower two (with volatility differing by a factor of six), respectively, form 'natural pairs.' We see many similarities, but also numerous systematic and significant differences. Specifically, in the lower pair comparing VOLATILITY and BOTH, we see that the share of subjects perceiving the low-volatility asset as the riskier one is always higher in BOTH than in VOLATILITY. Most likely this can be attributed to the task of assessing comparative risk being more demanding when the scaling is different on the vertical axis. In addition, also the share of subjects who were indifferent between the two assets is always higher in BOTH than in VOLATILITY – even though volatility is six times larger for one of the two assets displayed. As a consequence of more people being indifferent or considering the low-volatility asset as the riskier one, the

[2] For these models perceived risk is substituted by 16 dummy variables for each possible Asset×Scale interaction term (eight assets presented in two different scales, NARROW and WIDE). Corresponding Wald tests are provided in Table A11.

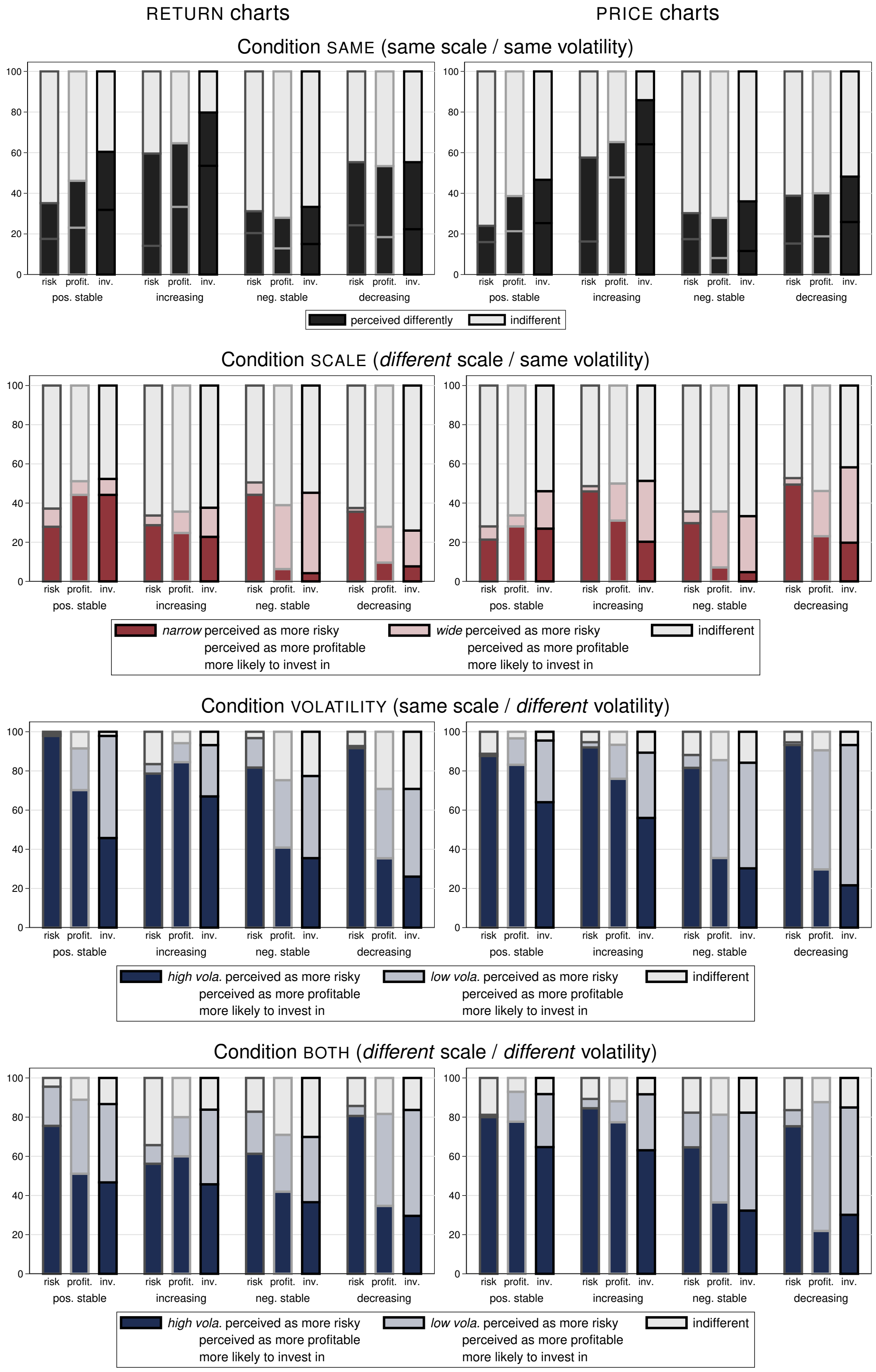

Figure B2: **Perceived riskiness, perceived profitability, and investment propensity in Task II.** This figure is identical to Fig. 7 in the main text.

Table B2: **Determinants of investment behavior**. The table presents the estimated coefficients of subject-fixed effects regressions with *investment propensity* (1 = very likely to invest, 7 = not likely to invest) as the dependent variable. The assets' average return and volatility, as well as subjects' perceived risk and return expectations act as independent variables. In Model (4), (4a), and (4b), perceived risk is substituted by 16 dummy variables for each possible Asset×Scale interaction term (eight assets presented in two different scales, NARROW and WIDE).

	Dependent variable: *Investment propensity*					
	Pooled Data				RETURNS	PRICES
	(1)	(2)	(3)	(4)	(4a)	(4b)
Hist. Return	0.438***		0.312***	0.395***	0.368***	0.376***
	(0.019)		(0.017)	(0.027)	(0.036)	(0.040)
Hist. Volatility	−0.156***		−0.026*	−0.159***	−0.235***	−0.106***
	(0.016)		(0.015)	(0.023)	(0.028)	(0.035)
Risk Perception		−0.413***	−0.413***			
		(0.015)	(0.015)			
Subj. Exp. Return (1y)		0.000	0.000	0.001	0.024***	0.001
		(0.001)	(0.001)	(0.001)	(0.009)	(0.001)
Subj. Exp. Return (5y)		0.030***	0.030***	0.036***	0.050***	0.043***
		(0.003)	(0.003)	(0.004)	(0.008)	(0.005)
Constant	0.561	3.934*	2.886	0.321	1.320	−0.627
	(2.560)	(2.238)	(2.240)	(2.518)	(3.164)	(3.739)
Subject-fixed effects	Yes	Yes	Yes	Yes	Yes	Yes
Asset dummies	Yes	Yes	Yes	–	–	–
Asset×Scale interaction	No	No	No	Yes	Yes	Yes
Observations	3,088	3,080	3,080	3,080	1,544	1,536
Adj. R^2	0.597	0.692	0.692	0.611	0.702	0.556

share of subjects identifying the high-volatility asset as the riskier one is significantly lower ($p < 0.05$) in BOTH than in VOLATILITY in 6 out of 8 pairwise tests.

When comparing the upper two panels, displaying conditions SAME and SCALE, we observe a high share of 40% to 70% of 'indifferent' choices between the two assets (who actually have the same volatility and trend and thus very similar or identical characteristics). However, that leaves up to 60% who see one of the two assets as less risky. Especially for the INCREASING trend subjects often express a preference for one asset – mostly for the one shown with the narrow scale in Condition SCALE.

When comparing the *perceived profitability* assessments in Fig. B2 vertically – i.e., we compare the same trends across the four different conditions (SAME, SCALE, VOLATILITY, and BOTH) – the upper two (with equal volatility) and the lower two (with volatility differing by a factor of six), respectively, again form 'natural pairs.' While we mostly see similarities, the differences are much less pronounced than in the risk assessment task: in the upper pair of panels we observe comparable shares of 'indifferent' choices with minor exceptions, e.g. for assets with INCREASING trend. For the lower pair, comparing VOLATILITY and BOTH, we see the choices to be quite similar as well: in the POSITIVE STABLE and INCREASING trends the

majority of subjects consider the more volatile asset to be more profitable (both for RETURN as well as PRICE charts), while for the rightmost two bars, displaying NEGATIVE STABLE and DECREASING trends, large shares (mostly more than 50%) of subjects stating a preference considering the low-volatility asset as the more profitable one. We conjecture that both patterns are mostly due to the fact that during upward trends, high volatility (and thus larger bars) are welcome, while during downward trends, smaller bars (hence, smaller price drops) are preferred. Thus, the display mode in conjunction with the prevailing price trend defines which assets subjects consider as more profitable.

B.4 Consistency across Tasks I and II

In the main text we consider risk perception in Tasks I and II separately. However, we also want to explore whether subjects are consistent across tasks.

There are 167 observations in our data where a subject sees the same assets with NARROW *and* WIDE scalings in Task I and also in a comparison of Condition SCALE in Task II. For these cases we find a small but significant correlation (Spearman's rank correlation coefficient $= 0.18$, $p < 0.05$) in the sense that perceiving risk with a NARROW scale as higher in the individual assessment is related to also perceiving the NARROW-scaled asset as more risky in pairwise comparisons. In addition, we estimated probabilities of perceiving the NARROW-scaled asset as more risky by running a probit model with the sign of the difference in risk perception from individual assessments as the independent variable. Results are plotted in Fig. B3. We see that with a positive difference (i.e. perceiving the NARROW-scaled asset as more risky in the individual assessment), the probability of also perceiving the NARROW-scaled asset as more risky in the pairwise comparison is significantly larger than .5 and is significantly larger than with a negative difference in the individual assessment.

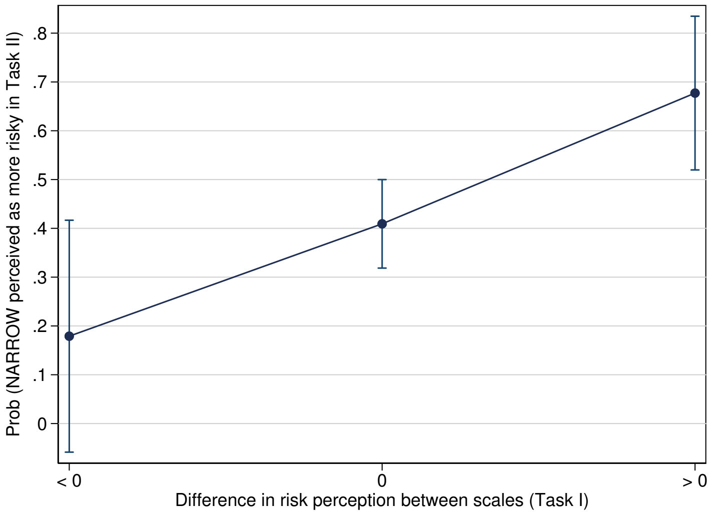

Figure B3: **Predicted probabilities of perceiving the narrow-scaled asset as more risky.** This figure shows the predicted probabilities and 95%-confidence intervals of perceiving the NARROW-scaled asset as more risky in Condition SCALE of Task II (different scale, same volatility) depending on the within-subject sign of the difference in perceived risk in Task I (NARROW minus WIDE). Probabilities are estimated from a probit model with subject-fixed effects and clustered standard errors. $N = 167$.

C Experimental Instructions[3]

General Instructions

Dear Participants!

Welcome to the experiment. From now on, please refrain from talking to other participants.

If you have any questions about the procedure or the instructions, please raise your hand. Your question will be answered in private. The whole experiment and analysis will be conducted anonymously.

This experiment consists of 6 parts in which you can earn money and a questionnaire.

Instructions Single Chart

Suppose you want to invest 5000 euros.

In the following, you will be presented with eight bar charts *(price charts)*, each showing the returns *(price development)* of a hypothetical asset in the past 10 years *as well as the current price.* (The return is defined as the percentage change of the price in one year.)

For each of these assets, we ask you to assess the following values:

- the asset's risk,
- the probability with which you would invest in this asset,
- the return *(price)* of the asset in the following year *(in a year)*,
- the (average) return *(price)* of the asset in the following five years *(in five years)*.

(!) Note that the scale of the charts may vary.

Instructions Compare Charts

Suppose you want to invest 5000 euros.

On the following eight pages, you will be presented with two bar charts *(price charts)* per page, which each show the returns *(price development)* in the past 10 years *(as well as the current price)*. (The return is defined as the percentage change of the price in one year.)

For each of these combinations you will be asked to compare the two shown assets on the basis of the following characteristics:

- the assets' risks,

[3] The experiment was conducted in German. The following instructions have been translated to English. The German instructions are available upon request. Text parts in *italics* are only shown when subjects are presented with price charts; text parts in standard font but in parentheses only refer to the parts in which subjects are presented with return charts.

- the assets' return opportunities,
- in which asset you would rather invest.

(**!**) Note that the scale of the charts may vary.

Payment: At the end of the experiment one of the eight rounds will be drawn randomly. The asset, which you chose in this round (that is, the one you would rather invest in), is relevant for your payment. If you did not decide to invest in one of the two assets in this round, one of the two will be chosen randomly. One of the ten past returns of this asset *(calculated as the percentage change of the price in one year)* will be chosen randomly.
You receive 5 € × (1 + 2 × this return.)

Example: Suppose the chosen return of the asset you would rather invest in is 10%. Then your payment is 5 € $\times(1 + 2 \times 0.10) = 6$ €.

D Examples of the decision screens

Task Ia

Teil 1: Runde 1 von 8

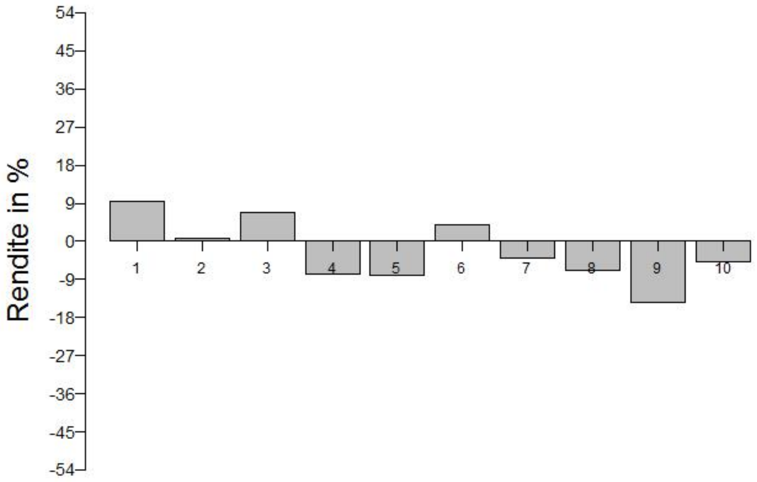

		1	2	3	4	5	6	7	
Für wie riskant halten Sie dieses Wertpapier?	gar nicht riskant	○	○	○	○	○	○	○	sehr riskant
Wie wahrscheinlich ist es, dass Sie in dieses Wertpapier investieren?	gar nicht wahrscheinlich	○	○	○	○	○	○	○	sehr wahrscheinlich

Wie hoch schätzen Sie die Rendite des Wertpapiers im folgenden Jahr? [ ] %

Wie hoch schätzen Sie die durchschnittliche Rendite des Wertpapiers in den folgenden fünf Jahren? [ ] %

Weiter

Task Ib

Teil 3: Runde 1 von 8

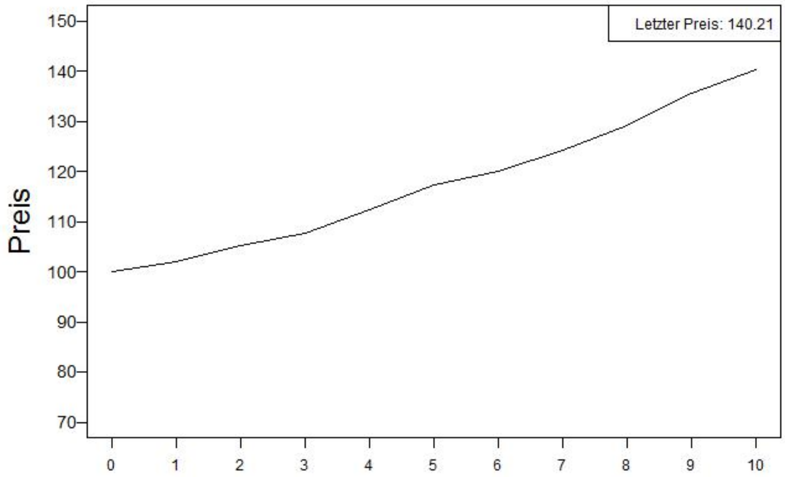

		1	2	3	4	5	6	7	
Für wie riskant halten Sie dieses Wertpapier?	gar nicht riskant	○	○	○	○	○	○	○	sehr riskant
Wie wahrscheinlich ist es, dass Sie in dieses Wertpapier investieren?	gar nicht wahrscheinlich	○	○	○	○	○	○	○	sehr wahrscheinlich

Wie hoch schätzen Sie den Preis des Wertpapiers in einem Jahr? [ ] Euro

Wie hoch schätzen Sie den Preis des Wertpapiers in fünf Jahren? [ ] Euro

Weiter

Task IIa

Teil 2: Runde 3 von 8

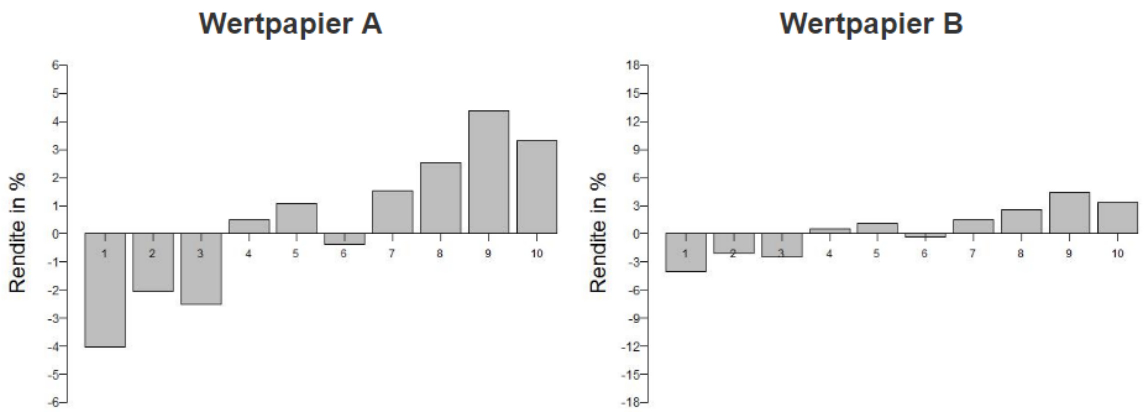

Welches Wertpapier halten Sie für riskanter?

○ Wertpapier A ○ beide gleich ○ Wertpapier B

Welches Wertpapier halten Sie für ertragsreicher?

○ Wertpapier A ○ beide gleich ○ Wertpapier B

In welches Wertpapier würden Sie eher investieren?

○ Wertpapier A ○ beide gleich ○ Wertpapier B

Weiter

Task IIb

Teil 4: Runde 2 von 8

Wertpapier A

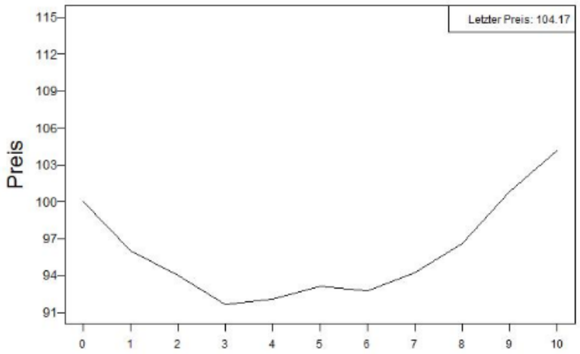

Wertpapier B

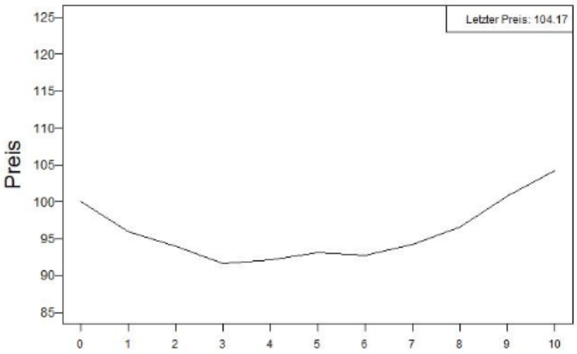

Welches Wertpapier halten Sie für ertragsreicher?

○ Wertpapier A ○ beide gleich ○ Wertpapier B

Welches Wertpapier halten Sie für riskanter?

○ Wertpapier A ○ beide gleich ○ Wertpapier B

In welches Wertpapier würden Sie eher investieren?

○ Wertpapier A ○ beide gleich ○ Wertpapier B

Weiter

E Examples of return and price chart comparisons in Task II

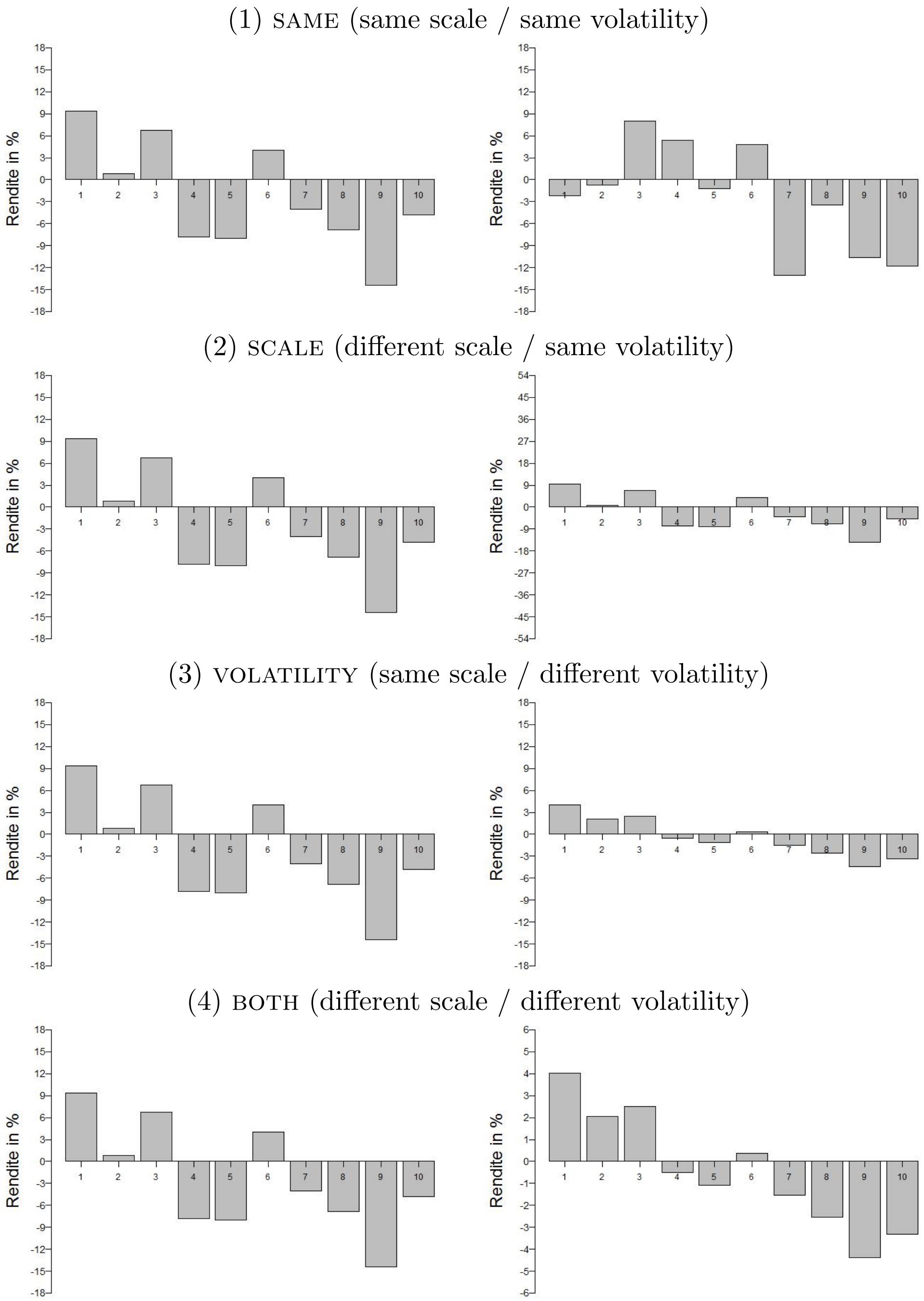

Figure E1: **Example of the treatment variation in volatility and scale in Task II.** This figure shows examples of the four conditions in Task II for the HIGH-volatility asset with trend DECREASING and presentation format RETURN.

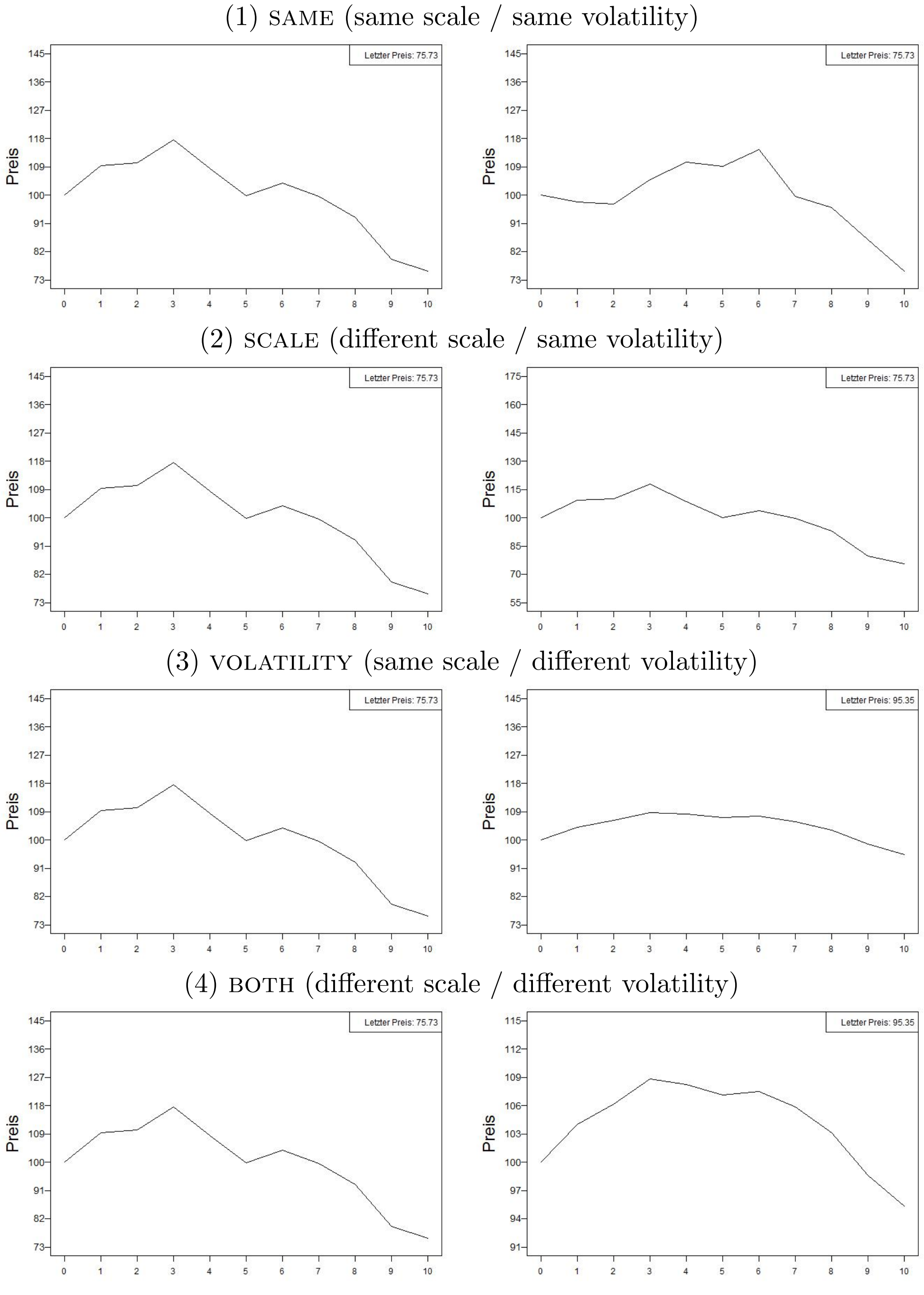

Figure E2: **Example of the treatment variation in volatility and scale in Task II.** This figure shows examples of the four conditions in Task II for the HIGH-volatility asset with trend DECREASING and presentation format PRICE.

References

Anzoni, Laura, Stefan Zeisberger. 2016. What is risk? How investors perceive risk in return distributions. Working Paper.

Huber, Jürgen, Stefan Palan, Stefan Zeisberger. 2018. Does investor risk perception drive asset prices in markets? Experimental evidence. Working Paper.

Jia, Jianmin, James S Dyer, John C Butler. 1999. Measures of perceived risk. *Management Science* **45**(4) 519–532.

Kaufmann, Christine, Martin Weber, Emily Celia Haisley. 2013. The role of experience sampling and graphical displays on one's investment risk appetite. *Management Science* **59** 323–340.

Nosić, Alen, Martin Weber. 2010. How riskily do I invest? The role of risk attitudes, risk perceptions, and overconfidence. *Decision Analysis* **7**(3) 282–301.

Sarin, Rakesh K, Martin Weber. 1993. Risk-value models. *European Journal of Operational Research* **70**(2) 135–149.

Unser, Matthias. 2000. Lower partial moments as measures of perceived risk: An experimental study. *Journal of Economic Psychology* **21**(3) 253–280.

Veld, Chris, Yulia V Veld-Merkoulova. 2008. The risk perceptions of individual investors. *Journal of Economic Psychology* **29**(2) 226–252.
